# H_2_S and NO cooperatively regulate vascular tone by activating a neuroendocrine HNO–TRPA1–CGRP signalling pathway

**DOI:** 10.1038/ncomms5381

**Published:** 2014-07-15

**Authors:** Mirjam Eberhardt, Maria Dux, Barbara Namer, Jan Miljkovic, Nada Cordasic, Christine Will, Tatjana I. Kichko, Jeanne de la Roche, Michael Fischer, Sebastián A. Suárez, Damian Bikiel, Karola Dorsch, Andreas Leffler, Alexandru Babes, Angelika Lampert, Jochen K. Lennerz, Johannes Jacobi, Marcelo A. Martí, Fabio Doctorovich, Edward D. Högestätt, Peter M. Zygmunt, Ivana Ivanovic-Burmazovic, Karl Messlinger, Peter Reeh, Milos R. Filipovic

**Affiliations:** 1 Department of Chemistry and Pharmacy, Friedrich-Alexander University Erlangen-Nuremberg, Egerlandstrasse 1, 91058 Erlangen, Germany; 2 Institute of Physiology and Pathophysiology Friedrich-Alexander University Erlangen-Nuremberg, Universitaetsstrasse 17, 91054 Erlangen, Germany; 3 Department of Anesthesiology and Intensive Care, Hannover Medical School, Carl-Neuberg-Strasse 1, 30625 Hannover, Germany; 4 Department of Physiology, University of Szeged, Dóm tér 10, H-6720 Szeged, Hungary; 5 Department of Nephrology and Hypertension, University of Erlangen-Nuremberg, Krankenhausstrasse 12, 91054 Erlangen, Germany; 6 Department of Pharmacology, University of Cambridge, Tennis Court Road, Cambridge CB1 2PD, UK; 7 Departamento de Química Inorgánica, Analítica y Química Física/INQUIMAE-CONICET, Universidad de Buenos Aires, Ciudad Universitaria, Pab. II, C1428EHA, Buenos Aires, Argentina; 8 Institute of Pathology, University of Ulm, Albert-Einstein-Allee 23, 89070 Ulm, Germany; 9 Department of Anatomy, Physiology and Biophysics, Faculty of Biology, University of Bucharest, Splaiul Independentei 91-95, 050095 Bucharest, Romania; 10 Departamento de Química Biológica, Facultad de Ciencias Exactas y Naturales, Universidad de Buenos Aires, Ciudad Universitaria, Pab. II, C1428EHA, Buenos Aires, Argentina; 11 Clinical Chemistry & Pharmacology, Department of Laboratory Medicine, Lund University Hospital, SE-221 85 Lund, Sweden; 12 These authors contributed equally to this work; 13 Present address: Institute of Physiology, RWTH Aachen University, Pauwelsstr. 30, 52074 Aachen, Germany

## Abstract

Nitroxyl (HNO) is a redox sibling of nitric oxide (NO) that targets distinct signalling pathways with pharmacological endpoints of high significance in the treatment of heart failure. Beneficial HNO effects depend, in part, on its ability to release calcitonin gene-related peptide (CGRP) through an unidentified mechanism. Here we propose that HNO is generated as a result of the reaction of the two gasotransmitters NO and H_2_S. We show that H_2_S and NO production colocalizes with transient receptor potential channel A1 (TRPA1), and that HNO activates the sensory chemoreceptor channel TRPA1 via formation of amino-terminal disulphide bonds, which results in sustained calcium influx. As a consequence, CGRP is released, which induces local and systemic vasodilation. H_2_S-evoked vasodilatatory effects largely depend on NO production and activation of HNO–TRPA1–CGRP pathway. We propose that this neuroendocrine HNO–TRPA1–CGRP signalling pathway constitutes an essential element for the control of vascular tone throughout the cardiovascular system.

## Abstract (web summary)

Nitric oxide (NO) and hydrogen sulphide (H_2_S) are two gaseous signalling molecules produced in tissues. Here the authors propose that NO and H_2_S react with each other to form nitroxyl (HNO), which activates the TRPA1 channel in nerve cells and triggers the release of the vasoactive peptide CGRP.

## Graphical abstract

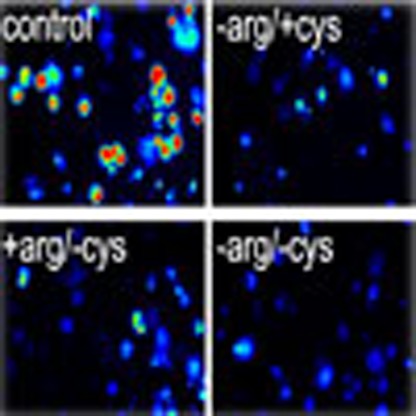

Nitroxyl, HNO, is the one-electron-reduced sibling of nitric oxide (NO) but follows an entirely separate signalling pathway^1^,^2^. HNO exerts systemic cardiovascular effects by releasing calcitonin gene-related peptide (CGRP) that combines general vasodilation with positive inotropic and lusitropic actions^3^,^4^. HNO donors, thus, provide a great promise for the treatment of heart failure, avoiding the problem of nitrate tolerance^4^,^5^,^6^. However, biochemical pathway(s) for HNO generation as well as the physiological mechanism of its CGRP releasing ability are still unknown, whereas abundant CGRP stores do exist inside ubiquitous sensory nerve fibres^7^,^8^,^9^. In this study, we aimed at understanding the actual biochemical pathway for HNO-induced CGRP release and at finding the actual source of HNO *in vivo*.

Recently, a new gaseous signalling molecule emerged, hydrogen sulphide (H_2_S), with physiological endpoints similar to that of NO^10^,^11^,^12^,^13^. Some studies even suggested that the effects of H_2_S and NO are mutually interdependent^13^,^14^,^15^,^16^. Elegant work on vascular endothelium and smooth muscle recently proposed a parallel formation of NO and H_2_S, the former entering the guanylyl cyclase pathway, the latter inhibiting phosphodiesterase 5, together thus stabilizing the level of vasorelaxant cyclic GMP^14^. A direct reaction of NO with thiols is too slow to be of physiological relevance^17^,^18^, whereas a direct interaction between NO and H_2_S has not yet been studied. We hypothesized, however, that the two gasotransmitters could enter a redox reaction with each other, which results in HNO formation—a potential way to intracellular HNO generation, if the H_2_S and NO producing enzymes were co-expressed. Knowing that the primary targets for HNO are thiols^1^,^2^ and the reason for its cardioprotective effect is CGRP release^3^,^4^, we hypothesized that HNO acts as an endogenous agonist of transient receptor potential channel A1 (TRPA1), a potential target for H_2_S (ref. 19) that is expressed in sensory nerve fibres and activated by numerous endogenous metabolites and environmental irritants^20^,^21^,^22^ through covalent modification of particular cysteine residues^23^,^24^, leading to para- and/or endocrine CGRP release and to local or general vasodilation, respectively.

## Results

### HNO activates TRPA1 in cells and sensory neurons

**Figure 1:**
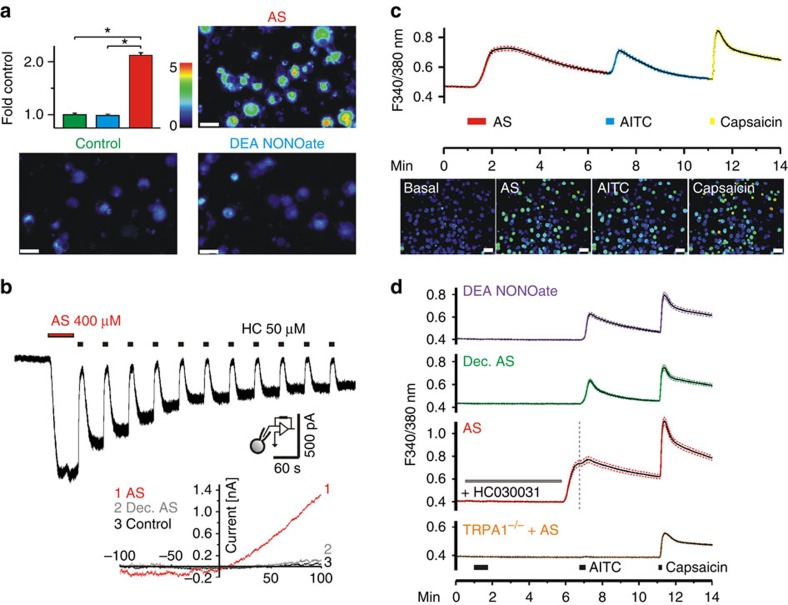
**HNO released by** AS **activates TRPA1 channels.** (**a**) Treatment with AS (300 μM) but not DEA NONOate (100 μM) increased fluorescence in sensory neurons (DRG) incubated with CuBOT1, a fluorescent dye for nitroxyl detection (analysis of variance following honestly significant difference *post hoc* test *P*<0.001; *P*=0.97, respectively; *n*=200 per group; scale bar, 40 μm). (**b**) AS (400 μM, 60 s) evoked inward currents in Chinese hamster ovary cells (*n*=7) expressing mTRPA1, which could be repeatedly blocked by HC030031 (50 μM, 10 s). Inset: Ramp currents through TRPA1 before (control, black) and during application of either decomposed AS (grey) or 400 μM AS (red). (**c**) Ca^2+^ influx (mean±s.e.m.) in DRG neurons of C57Bl/6 mice treated with AS (300 μM, 45 s), AITC (100 μM, 20 s) and capsaicin (0.3 μM, 10 s at 4-min intervals) and corresponding representative pseudocolor images (scale bar, 80 μm). (**d**) AS effects are abolished by application of HC030031 (50 μM) or decomposed AS and absent in TRPA1^−/−^ mice. DEA NONOate (100 μM, 45 s) caused no effect. Data represent mean±s.e.m., *n*=300 DRG neurons for each group.

The effects of HNO on Chinese hamster ovary cells stably expressing mTRPA1 were examined by whole-cell voltage clamp. Angeli’s salt (AS) was used as a donor of HNO (Fig. 1). AS decomposes in neutral buffer to give equimolar amounts of nitrite and HNO with a half-life of about 5 min at 37 °C (ref. 25). In addition, HNO dimerizes rapidly (8 × 10^6^ M^−1^ s^−1^) (ref. 25) with elimination of water to give inert N_2_O. Considering the inherent delays in the various experimental set-ups, this implies that the actual concentration of HNO in physiological buffer at the time of measurement was lower (less than half) than that of AS initially dissolved and explains a relatively high dose of AS required to induce an effect. Sixty-second exposures to (initially) 400 μM AS-evoked large sustained inward currents, which could be repeatedly blocked by the TRPA1 channel antagonist HC030031 (Fig. 1b). Control treatment with decomposed AS showed no effect (Supplementary Fig. 1). Measuring ramp currents (−100 to +100 mV in 500 ms), we observed that AS induced an outwardly rectifying current–voltage relationship (Fig. 1b), while decomposed AS was ineffective.

To confirm the observed effects in native sensory neurons, Ca^2+^ influx was measured in cultured, Fura-2-stained dorsal root ganglion (DRG) neurons of wild-type and TRPA1^−/−^ mice. Forty-five-second exposures to AS caused an increase in intracellular Ca^2+^ in 28 % of wild-type cells (Fig. 1c). The correlation of the cells responding to AS with responses to the prototypic TRPA1 agonist allyl isothiocyanate (AITC 100 μM, *r*=0.625) strongly implied the involvement of TRPA1. This was supported by a complete block of AS-evoked Ca^2+^ increase under HC030031 (Fig. 1d). Wash-out of HC030031 caused an immediate recovery of the previously suppressed AS response, similar to the repeated inhibition and recovery observed in patch clamp experiments (Fig. 1b), suggesting that the activating modifications induced by HNO treatment were sustained. In cells prepared from TRPA1^−/−^ mice, AS, like AITC, had no effect (Fig. 1d), proving that TRPA1 was the receptor channel activated by HNO. In wild-type mice, there was no effect of decomposed AS (Fig. 1d) or of AS applied in Ca^2+^-free external solution excluding intracellular store depletion (Fig. 1b).

The use of a pure NO donor (DEA NONOate) at doses similar to HNO had no effect on intracellular Ca^2+^ (Fig. 1d). However, high concentrations of NO did induce slow Ca^2+^ entry, indicative of non-physiological formation of high levels of N_2_O_3_ acting as an *S*-nitrosating agent^17^ (Supplementary Fig. 2a). In fact, the use of a direct *S*-nitrosating agent, SNAP, induced reversible Ca^2+^ influx (at concentrations >1 mM), with the effect being quickly abolished when SNAP was removed (Supplementary Fig. 2b), clearly distinguishing it from sustained Ca^2+^ influx observed with AS.

### HNO-induced disulphide formation on TRPA1

**Figure 2:**
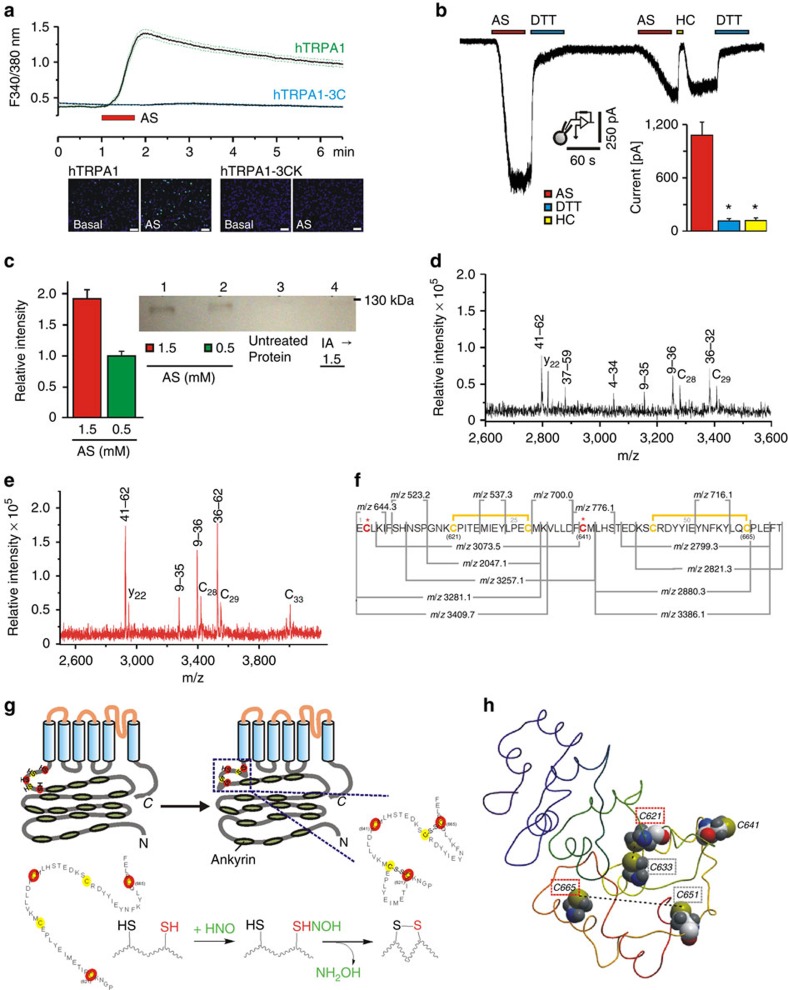
HNO activates TRPA1 via disulphide bond formation. (**a**) AS (300 μM, 45 s) increases intracellular Ca^2+^ in hTRPA1-transfected HEK cells but not in cells expressing hTRPA1-3C; mean±s.e.m., *n*=250 cells for each group. Representative pseudocolor images (scale bar, 100 μm). (**b**) AS-evoked inward currents (*n*=7) can be reversed by application of DTT (5 mM, 60 s), or temporarily blocked by HC030031 (50 μM, 10 s). Inset: AS (400 μM)-evoked peak inward currents are significantly reduced by HC030031 or DTT (analysis of variance following honestly significant difference *post hoc* test, *P*<0.001 each; *n*=7; error bars represent s.e.m.). (**c**) Detection of disulphide bond formation on purified mTRPA1 channel protein (100 μM) treated with AS, using modified biotin-switch assay. Lane 1: TRPA1 treated with 1.5 mM AS, lane 2: TRPA1 treated with 0.5 mM AS, lane 3: untreated protein, lane 4: TRPA1 pretreated with IA and then treated with 1.5 mM AS. (**d**,**e**) MALDI-TOF MS of AS-treated TRPA1 synthetic peptide. Peptide fragments containing cysteine residues differ in AS-treated (black, **d**) and control (red, **e**) samples. The same fragmenting pattern is observed in both cases but with *m/z* of the AS-treated fragments being shifted towards lower masses (by *m/z* 116=2IA+2H), indicating formation of disulphides. (**f**) Amino-acid sequence of synthetic peptide used in the study to mimic the part of hTRPA1 N terminus with critical, that is, activating cysteines and a rationale for deciphering disulphide bond positions based on observed fragments. Yellow marked cysteine residues form disulphide bonds and red cysteine residues are found to be modified by IA even after exposure to AS. (**g**) Schematic model of TRPA1 with cysteine-rich region (red dots) and formation of disulphide bonds causing major conformational changes. Chemical structure (bottom) of two cysteine-SH residues reacting with HNO to form hydroxylamine (NH_2_OH) and a disulphide bond and causing conformational change. (**h**) *Ab initio* model of the 200 amino acid long polypeptide chain of the N terminus of hTRPA1 displaying five essential cysteine residues and two indicated disulphides (dotted lines).

hTRPA1 is rich in cysteine residues and C621, C641 and C665 of the N terminus are responsible for electrophilic activation^23^. To test whether these specific cysteine residues play a role, experiments were performed on HEK293 cells expressing hTRPA1 and cysteine- and lysine-neutralized mutants, hTRPA1-C621S/C641S/C665S (hTRPA1-3C) and hTRPA1-C621S/C641S/C665S/K710R (hTRPA1-3CK). AS had no effect on non-transfected HEK cells but induced large responses in hTRPA1-transfected cells. Cells expressing hTRPA1-3CK or hTRPA1-3C did not respond to AS at all (Fig. 2a). Although many TRPA1 agonists at higher concentrations also activate TRPV1 (ref. 26), no responses could be observed in experiments on hTRPV1-transfected HEK cells (Supplementary Fig. 2c).

Providing that the observed effects originate from disulphide formation, the reducing agent dithiothreitol (DTT) should interfere with the outlasting TRPA1 responses. Indeed, the decay of the AS responses was considerably accelerated when 5 mM DTT was externally applied, and the downward inflection upon DTT onset almost restored intracellular Ca^2+^ to baseline level within 10 min (Supplementary Fig. 3). HC030031 applied after AS caused similar effects, however, after its removal, a rebound rise in intracellular Ca^2+^ occurred, confirming a temporary block in presence of a sustained TRPA1 modification by HNO.

The outlasting AS-induced inward currents were instantly reversed by the administration of 5 mM DTT and did not recur upon its washout (Fig. 2b). When the cells were subsequently re-exposed to AS, the responses were smaller (62.5% of first response). These currents could be blocked by HC030031 only for the duration of its application, and DTT again deactivated the recurred inward current completely.

Formation of disulphides upon HNO stimulation of TRPA1 was further supported using a modified biotin-switch assay (Fig. 2c) on V5-poly-His-tagged mTRPA1 expressed and purified from HEK cells (Supplementary Fig. 4a–d). The protein that was exposed to HNO provided positive staining in a concentration-dependent manner while the control or the iodoacetamide (IA)-pretreated protein exposed to HNO showed no disulphide bond formation (Fig. 2c).

To identify the reaction site, a custom-made synthetic peptide consisting of 64 amino acids of the hTRPA1 N terminus, including the three critical and three neighbouring cysteine residues, was analysed by MALDI-TOF mass spectrometry (MS) (Fig. 2d,e, Supplementary Fig. 4e). MS analysis revealed disulphide formation between the critical Cys 621 and the neighbouring Cys 633 as well as between Cys 651 and the critical Cys 665 (Fig. 2f).

Formation of disulphides by HNO would go step-wise, with initial formation of a (hydroxyamino)sulfanyl derivative at critical cysteine residues and then fast subsequent reaction with another cysteine in vicinity, leading to substantial allosteric deformation and channel opening (Fig. 2g). Such disulphide bonds may account for the observed slow deactivation of TRPA1 after AS treatment. *Ab initio* structure prediction of a 200 amino acid long N-terminal sequence covering important cysteine residues (Fig. 2h) revealed that the cysteine residues 665 and 651 are connected by a flexible loop and could easily get into each other’s vicinity, a condition that would facilitate disulphide bond formation. In addition, disulphide bond formation is facilitated between the Cys 633 and 621, where the atom distance is estimated to be 4.4 Å.

### TRPA1 is responsible for HNO-induced release of CGRP

**Figure 3:**
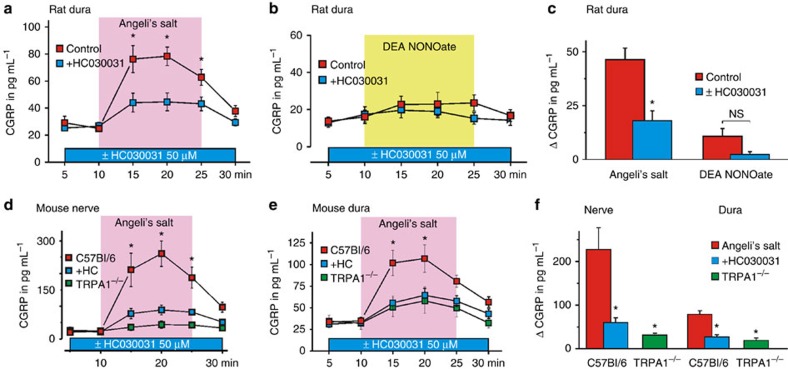
HNO activation of TRPA1 releases CGRP. (**a**) AS (500 nmol per skull half) reversibly induced CGRP release from rat cranial *dura mater*, which was diminished by application of HC030031 (Wilcoxon matched pairs tests, *P=0.008, *n*=9 repeated measures ANOVA and HSD post hoc tests, *P<0.05, *n* given in the figure). (**b**,**c**) DEA NONOate (333 nmol per skull half) did not stimulate CGRP release from rat *dura mater encephali*, while AS (500 nmol)-induced CGRP release was diminished by application of HC030031 (analysis of variance (ANOVA) honestly significant difference (HSD) *post hoc* test, *P<0.001, *n*=9 each). (**d**) CGRP release from sciatic nerve of C57Bl/6 mice is stimulated by AS (250 nmol). Co-application of HC030031 (50 μM) reduces AS-stimulated CGRP release (*U*-test, *P*≤0.02; *n*=7) while the AS effect is abolished in TRPA1^−/−^ mice. (**e**) CGRP release from mouse *dura mater encephali* of C57Bl/6 mice is activated by AS (250 nmol per skull half) and diminished by HC030031 (50 μM) (Wilcoxon matched pairs test, *P*=0.01 each, *n*=8); CGRP release is also markedly reduced in preparations of TRPA1^−/−^ mice treated with AS (250 nmol per skull half). (**f**) Histogram showing the maximal CGRP released from C57Bl/6 and TRPA1^−/−^ mouse sciatic nerve and *dura mater* upon stimulation with AS. AS effects were reduced by HC030031 (50 μM) and absent in TRPA1^−/−^ (ANOVA HSD *post hoc* tests, *P≤0.01 compared with AS-treated C57Bl/6; all error bars represent s.e.m.).

Next we tested how HNO-induced TRPA1 activation affects CGRP release from isolated tissues. CGRP is typically released from polymodal A-delta and C-fiber afferents^27^. At first, the release of CGRP from rat dura mater exposed in a hemisected skull preparation was measured in 5-min samples of incubation fluid using enzyme immunoassays (Fig. 3a–c). Exposure to AS induced an increase in CGRP release (by 46.4±5.3 pg ml^−1^) while DEA NONOate was ineffective (Fig. 3b,c) confirming previous observations of distinct biochemical pathways that these two congeners enter downstream of nitric oxide synthase (NOS)^1^,^2^,^3^,^4^. HC030031 significantly inhibited the AS-evoked responses (18.1±4.6 pg ml^−1^, Fig. 3a,c).

We also added evidence from another species and innervation territory measuring CGRP release from sciatic nerve (Fig. 3d) and cranial dura mater (Fig. 3e) of wild-type and TRPA1^−/−^ mice. In both preparations, HC030031 had the same inhibitory effects (Fig. 3f) as observed in rat (Fig. 3a,c) but preparations taken from congenic TRPA1^−/−^ mice did not respond to AS stimulation at all (Fig. 3f). These results confirm that HNO-induced CGRP release is selectively mediated via TRPA1 receptor channels.

**Figure 4:**
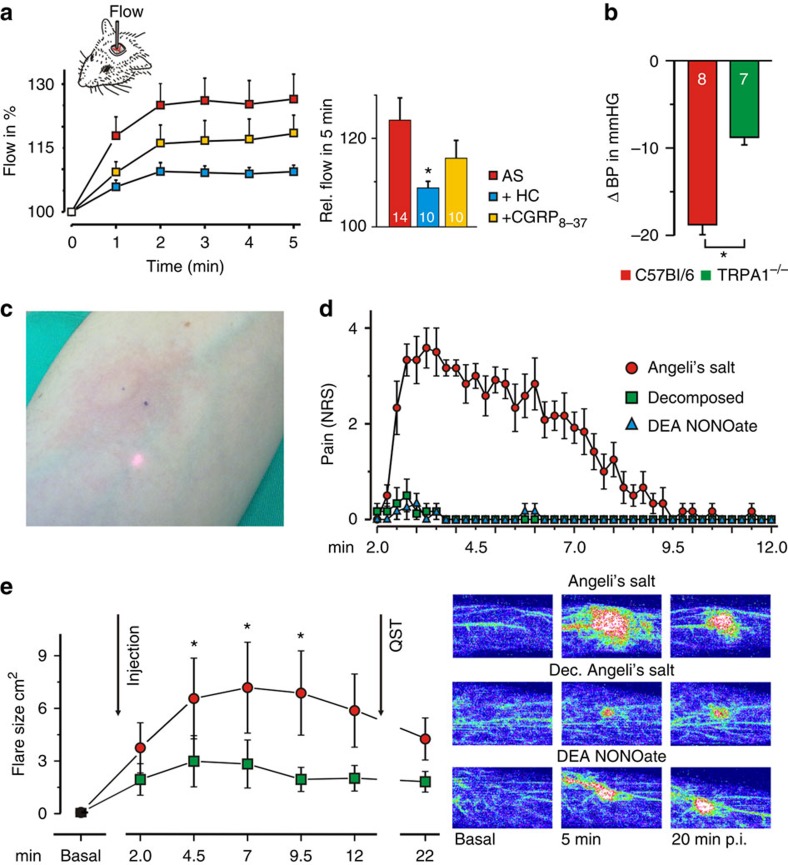
**Vasodilation by HNO-induced CGRP release involves TRPA1** ***in vivo***. (**a**) AS-evoked increase in meningeal blood flow measured in anaesthetized rats was significantly reduced by topical application of HC030031 (50 μM) (mean±s.e.m., repeated measures ANOVA and HSD post hoc tests, *P<0.05, *n* given in the figure). (**b**) AS-induced drop in MAP under anaesthesia was smaller in TRPA1^−/−^ than in C57Bl/6 mice (*P*<0.002; *U*-test; *n*=7). (**c**) Photograph of a subject’s volar forearm with noticeable axon-reflex erythema upon intradermal injection of AS (0.7 μmol). (**d**) Magnitude and time-course of AS (0.7 μmol)-evoked pain in human volunteers (*n*=6, 3 male and 3 female) on a numerical rating scale (NRS 0–10). Decomposed Angeli's salt and DEA NONOate (0.23 μmol) were used as controls. (**e**) Areas of increased superficial blood flow following injections of AS and decomposed AS as a control. QST indicates time point of quantitative sensory testing. Pseudocolor representations of laser Doppler scanned images of superficial blood flow evoked by injection of AS, DEA NONOate and decomposed AS (repeated measures analysis of variance, least significant difference *post hoc* tests; *P≤0.05; *n*=6; all error bars represent s.e.m.).

CGRP is a potent vasodilator contributing to overall cardiovascular homeostasis^7^. It is also released from trigeminal nerve fibres accompanying meningeal blood vessels and plays an established role in migraine^8^. In anaesthetized rats, topically applied AS (60 nmol) induced an increase in meningeal blood flow (recorded by laser Doppler flowmetry) by 24 %, while co-application of HC0300301 (50 μM) reduced this response by 64% (Fig. 4a). Co-application of CGRP receptor antagonist, CGRP_8–37_, also inhibited the AS-evoked dilatation, although not as effectively as TRPA1 channel blockade, most likely due to modification of the inhibitory peptide by HNO (Supplementary Fig. 5).

To assess to what extent the systemic hypotensive effect of HNO depends on TRPA1, the mean arterial blood pressure (MAP) of anesthetized mice was measured. Mice were injected intravenously with freshly dissolved AS (61 μg kg^−1^ body weight) in saline solution (pH 7.0). AS induced a drop of MAP by 18.8±1.2 mm Hg in wild type, but only 8.8±0.9 mm Hg in TRPA1^−/−^ mice (Fig. 4b), indicating that a significant proportion of the vasodilatory effect of HNO is mediated by TRPA1.

CGRP release from sensory nerve fibres does not necessarily require action potential discharge; subliminal depolarization is sufficient^9^. Nonetheless, strong activation of TRPA1-expressing peptidergic nerve fibres should evoke pain. This was tested in human volunteers by double-blinded intracutaneos injection of AS (0.7 μmol, Fig. 4c). Injections of AS caused an immediate burning pain declining over ~7.5 min with a mean maximum rating of 3.6±0.4 (on a numerical rating scale 0–10, *n*=6). Decomposed AS and DEA NONOate were neither rated as painful nor as itching (Fig. 4d). Although TRPA1 is not activated by noxious heat in heterologous expression systems, its activation by cinnamaldehyde causes heat hyperalgesia in humans^28^. In our experiments, injection of AS but not of DEA NONOate or decomposed AS increased the pain ratings (from 2.6±0.6 to 4.3±0.7, *P*<0.001, analysis of variance and least significant difference) in response to noxious heat (5 s, 47 °C). In addition, injection of AS into the skin of the volar forearm induced a large axon-reflex erythema (Fig. 4c) visualized by laser Doppler scanning of superficial blood flow (Fig. 4e, Supplementary Fig. 6a), which was not affected by the pre-administration of Clemastine, an H1 histaminic receptor blocker, indicating that the AS-induced vasodilation is not due to mast cell degranulation (Supplementary Fig. 6b,c).

### Generation of HNO from NO and H_2_S

**Figure 5:**
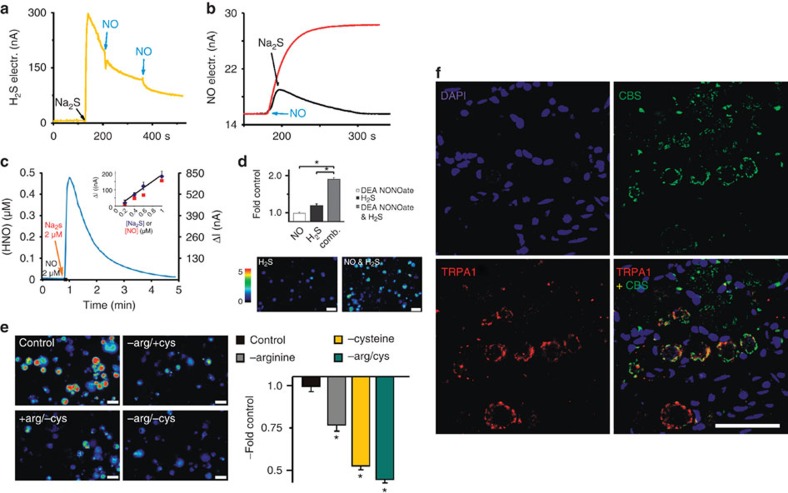
**HNO formation is NO and H**_**2**_**S dependent.** (**a**,**b**) H_2_S and NO react *in vitro* as observed by the drop of H_2_S electrode response upon subsequent additions of NO solution (**a**) and by the drop of NO electrode response when H_2_S was injected into solution containing DEA NONOate (**b**). (**c**) Amperometric signal of the HNO-selective electrode after addition of H_2_S (2 μM) to a solution of 2 μM NO (left axis: (HNO) after calibration, right axis: measured current). Inset: Signal peak versus H_2_S (blue) and NO (red) concentration, while the other reactant concentration is maintained constant and in excess. (**d**) Treatment with DEA NONOate (100 μM) and H_2_S (100 μM) for 15 min increased fluorescence in DRG neurons incubated with CuBOT1. (Analysis of variance (ANOVA) honestly significant difference (HSD) *post hoc* test; *P*<0.001, respectively, *n*=116 each, scale bar, 80 μm). (**e**) Basal fluorescence of the HNO sensor in sensory neurons was reduced by arginine and cysteine depletion, substrates for endogenous NO and H_2_S production (ANOVA HSD *post hoc* test; *P<0.001, treated versus control respectively; *n*=150; all error bars represent s.e.m., scale bar, 50 μm). (**f**) Confocal images of a rat trigeminal ganglion section immunohistochemically double stained with antibodies against TRPA1 (Cy3, red) and CBS (FITC, green) combined with nuclear 4′,6-diamidino-2-phenylindole hydrochloride (DAPI) staining (blue; scale bar, 50 μm).

The cross-talk of H_2_S and NO has been suggested^14^,^15^,^16^ but the actual direct reaction of NO with H_2_S has not been studied before. The reaction of NO and H_2_S solutions was initially probed amperometrically using H_2_S and NO selective electrodes. The drop of the H_2_S electrode response upon addition of gaseous NO suggested immediate H_2_S consumption under aerobic conditions (Fig. 5a) and the apparent rate of this reaction remained unchanged even in the presence of 20-fold excess of glutathione in anoxic conditions (Supplementary Fig. 7a). In parallel, NO release from DEA NONOate was prevented when H_2_S was present in the solution (Fig. 5b). Both, the addition of NO to degassed H_2_S solution, or H_2_S to NO solution led to immediate sulphur/polysulphide formation suggesting that H_2_S was oxidized and consequently NO was reduced.

The ultimate proof for the *in vitro* HNO formation came from the recently developed HNO-selective electrode^29^. The results show that a rate of HNO production through an anaerobic reaction between NO and H_2_S is orders of magnitude faster than for any known HNO donor (Fig. 5c, Supplementary Fig. 7b). For example, 2 μM combination of each NO and H_2_S yields a peak HNO concentration of ca. 0.5 μM, similar to effects of almost three orders of magnitude higher AS concentration (1 mM, Fig. 5c).

Finally, HNO formation was also tested on cellular level using a HNO-selective fluorescent sensor^30^. Only the combination of applied NO and H_2_S provided strong fluorescence signals characteristic of HNO (Fig. 5d). Interestingly, a basal fluorescence of the untreated cultured neurons was observed, suggesting that some basal formation of HNO was constitutively present. Thus, we inhibited enzymatic NO and/or H_2_S production to see the effect on basal intracellular HNO level. In neurons, treatment for 2 h with either L-NMMA (inhibitor of NOS), oxamic acid (inhibitor of cystathionine beta synthase, CBS) or with the combination of both resulted in a dramatic decrease of the basal intracellular HNO signal (Supplementary Fig. 7c). DRG cells deprived of arginine, cysteine or both for 2 h (Fig. 5e) provided the same results, suggesting that the majority of intracellularly produced HNO originated from the interaction of NO and H_2_S that are constitutively produced.

Both NO and H_2_S are gasotransmitters and as such, could freely diffuse into nerve fibres^31^ to deliver paracrine signals. In addition, upon adequate stimulation including Ca^2+^ influx, neurons may be able to produce HNO by themselves, modifying TRPA1 to become more sensitive or activated in a sustained way. Neuronal NOS has already been shown to colocalize with TRPA1 (ref. 32), but CBS has not been examined before. We thus focused on detecting CBS and TRPA1 in sensory neurons and fibres. TRPA1 immunoreactivity was preferably found in small and CBS in small and middle-sized trigeminal neurons, producing a yellow colour in the merged image where both were colocalized (Fig. 5f). High magnification shows the immune products in discrete, well-organized structures confirming a strong co-expression of the H_2_S-producing enzyme with TRPA1. In confocal images of cross-sections through the rat spinal trigeminal nucleus caudalis (Supplementary Fig. 7d), bundles of immunostained afferent nerve fibres were seen running through the trigeminal tract and into the superficial laminae of the nucleus. Most of the nerve fibres show signals for both TRPA1 and CBS, producing the yellow colour in the merged image.

### HNO from NO and H_2_S activates TRPA1 to cause CGRP release

While we show that NO provided by a pure NO donor does not directly activate TRPA1 (Supplementary Fig. 2a), we also tested H_2_S on cultured DRG neurons and could not see any significant change in intracellular Ca^2+^ levels when using 100–500 μM (Supplementary Fig. 8a). Prolonged exposures to H_2_S did, however, induce channel activation in a cysteine-dependent manner (Supplementary Fig. 8b), but this treatment inevitably leads to inhibition of cell respiration^33^ and transient ROS formation^34^ both of which could activate TRPA1 (refs 35, 36) and cannot account for the physiological effects of H_2_S in low concentration.

**Figure 6:**
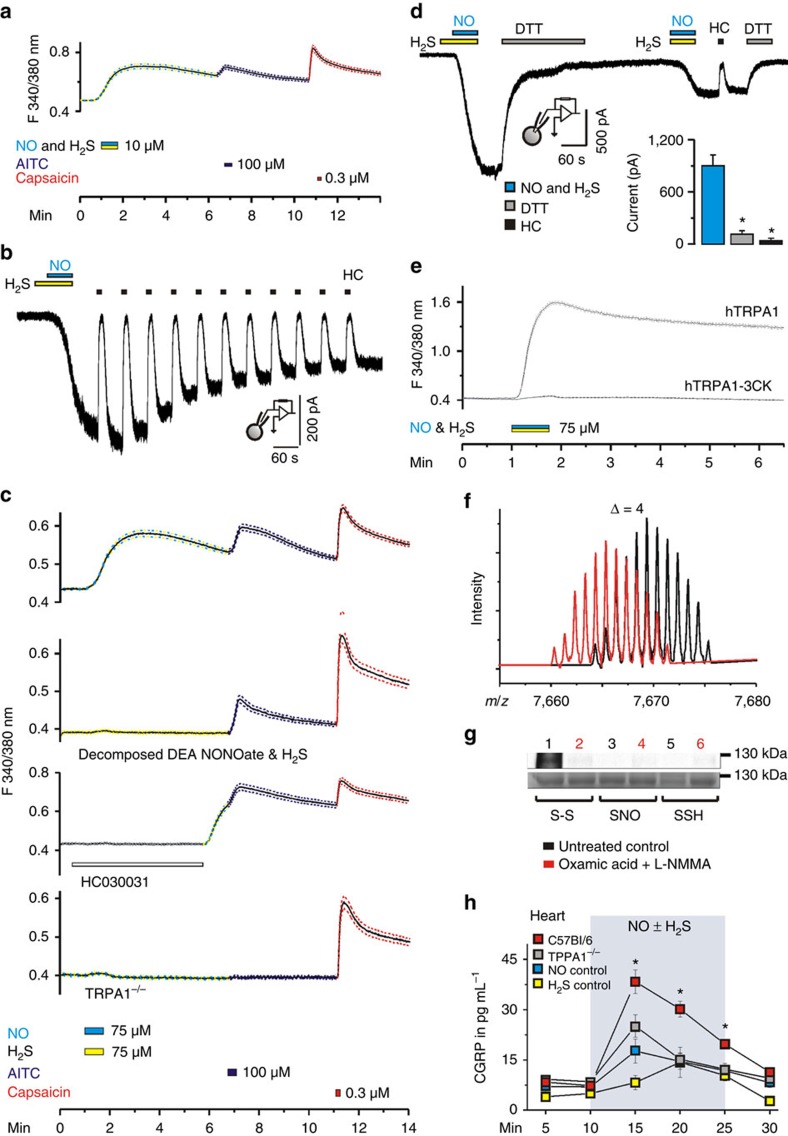
**HNO generated from NO and H**_**2**_**S activates TRPA1 to release CGRP.** (**a**) Combination of NO and H_2_S (10 μM each) induces increases of intracellular Ca^2+^ in DRG neurons of C57Bl/6 mice. AITC (100 μM, 20 s) and capsaicin (0.3 μM, 10 s at 4-min intervals) were used as controls. Data represent mean±s.e.m. (**b**) Similar to the currents evoked by AS (Fig. 1b), in mTRPA1-expressing Chinese hamster ovary cells, inward currents are induced as soon as DEA NONOate (75 μM) is added to H_2_S (75 μM). Currents (mean±s.e.m., *n*=7) can reversibly be blocked by HC030031 (50 μM, 10 s). (**c**) Combination of NO and H_2_S (75 μM each) induces reversible increases of intracellular calcium in Fura-2-stained DRG neurons of C57Bl/6 mice (AITC and capsaicin were used as controls as above). Responses were absent when DEA NONOate was decomposed before combined application with H_2_S and abolished following treatment with HC030031 (50 μM) or in TRPA1^−/−^ mice (mean±s.e.m.; *n*=375). (**d**) DTT (5 mM, 60 s) reversed NO+H_2_S-evoked inward currents (*n*=7), which could temporarily be blocked by HC030031. (**e**) Combination of NO and H_2_S (75 μM each) activates hTRPA1 increasing intracellular Ca^2+^ in transfected HEK cells but not in cells expressing hTRPA1-3CK; mean±s.e.m., *n*=250 cells each. (**f**) *m/z*=(−4) spectral shift of 64 amino acid long peptide treated with the combination of NO and H_2_S (red) compared with untreated peptide (black). (**g**) Detection of intramolecular disulphides (1–2), *S*-nitrosothiols (3–4) and *S*-sulfhydration (5–6) in TRPA1 isolated form DRG neurons (lanes 1, 3, 5) or DRG neurons treated with 2 mM combination of oxamic acid and L-NMMA for 12 h (lanes 2, 4, 6). Lower picture: total protein load. (**h**) CGRP release from hearts of mice induced by superfusion with 250 nmol NO and/or H_2_S. (ANOVA least significant difference *post hoc* test, *P*<0.003 for C57Bl/6 compared with either NO, H_2_S or TRPA1^−/−^, *n*>11 for TRPA1^−/−^ and C57Bl/6, *n*=6 for NO and H_2_S, error bars represent s.e.m.).

However, when combined, H_2_S and NO did induce dramatic effects. The effects of combined H_2_S and NO (10–75 μM) were scrutinized in the same way as previously for AS, using Ca^2+^ imaging and patch clamp, TRPA1 agonists and antagonists, knockouts and mutants and the outcomes were strikingly identical to what was observed with HNO stimulation, a clear specific activation of TRPA1 in sensory neurons through interaction with the critical N-terminal cysteines (Fig. 6a–d and Supplementary Fig. 9). Combination of 10 μM of each NO and H_2_S induced a similar maximal response as that observed with 75 μM concentrations (Fig. 6a,c).

H_2_S has been suggested to modify cysteine residues inducing formation of persulphides, however, a direct reaction of H_2_S with cysteine residues is chemically impossible unless in the presence of an oxidant^37^. Nonetheless, we used the purified N terminus of TRPA1 channels (amino acids 1–719) and treated it with DEA NONOate, H_2_S or both under hypoxic (2–5% O_2_) conditions to minimize the artifactual oxidation of cysteine residues. Following this, the protein was treated with IA to block all free cysteine residues, then exposed to DTT to remove eventual cysteine modifications and finally treated with Ellman’s reagent. If any of the following modifications *S*-nitrosation, persulphide or disulphide formation, were present it should have yielded a yellow product formation with characteristic visible spectral properties. However, only when the NO/H_2_S combination was used such a characteristic product was observed, confirming that neither NO nor H_2_S directly modify the channel (Supplementary Fig. 10a). Furthermore, a purified N terminus of TRPA1 mutant hTRPA1-C621S/C641/C665S (amino acids 1–719) did not show a positive Ellman’s reaction when exposed to the same treatments (Supplementary Fig. 10b). Using the synthetic model peptide as in Fig. 2, we confirmed this finding (Fig. 6f; Supplementary Fig. 10c) and could show that, when exposed to NO/H_2_S, the peptide gets modified, because the *m/z* shift by 4 implies that two disulphide bonds are formed as expected from our results with AS (Fig. 6f). Finally, we purified the TRPA1 channel by immunoprecipitation from dorsal root ganglia and compared the levels of endogenously present disulphides, *S*-nitrosothiols and persulphides in controls and ganglia treated with combination of NOS and CBS inhibitors. Only endogenously present disulphides were found and their level was significantly reduced by the treatment with L-NMMA and oxamic acid, suggesting that HNO is a constitutive endogenous regulator of TRPA1 activity (Fig. 6g).

Finally, we tested the ability of the NO/H_2_S combination to induce CGRP release from the isolated mouse heart (Fig. 6h, Supplementary Fig. 11a,b), because the heart is an important target organ of circulating CGRP^3^,^4^. CGRP is released from chemosensory primary afferent nerve fibres located in the epicardial surface of the heart, and human cardiomyocytes do express the receptor for CGRP (Supplementary Fig. 11c,d). We show that neither 250 nmol H_2_S nor NO have any significant effects on CGRP release. However, the combination of both proved to be a robust stimulant with CGRP peaking at 38±4 pg ml^−1^ in the effluate (Fig. 6h). While HC030031 reduced this effect, the failure of the TRPV1 blocker BCTC, as well as the use of TRPV1^−/−^ mice proved that TRPV1 is not involved in this process (Supplementary Fig. 11b). If hearts from TRPA1^−/−^ mice were used, the peak of the CGRP release was reduced and the stimulant effect lasted much shorter compared with congenic C57Bl/6 mice (Fig. 6h).

### H_2_S vasodilatory effects are NO and TRPA1 and CGRP dependent

**Figure 7:**
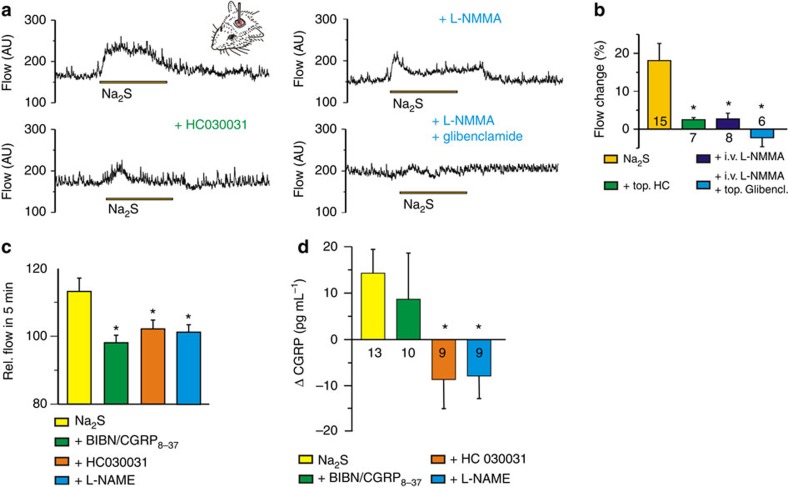
**Local neurovascular effects of H**_**2**_**S depend on NO and TRPA1 and CGRP.** (**a**) Original recordings of meningeal blood flow in anaesthetized rats using laser Doppler flowmetry: H_2_S (60 nmol), HC030031 (50 μM), i.v. L-NMMA (10 mg kg^−1^) and 1 mM glibenclamide with i.v. L-NMMA. (**b**) Mean values of flow increase (normalized to baseline) within 5 min after topical administration of H_2_S (ANOVA least significant difference (LSD) *post hoc* tests; *P<0.05, *n*=6). (**c**) Changes in brainstem blood flow upon i.v. injection of 70.2 μg kg^−1^ Na_2_S. The TRPA1 antagonist (HC030031), CGRP receptor antagonists (BIBN4096BS and CGRP_8–37_) and NOS inhibitor (L-NAME) were applied i.v. (**d**) Changes of CGRP levels in cerebrospinal fluid following the treatment shown in **c** (repeated measures ANOVA, LSD *post hoc* test; *n* given in the figure, *P<0.05 compared with Na_2_S, all error bars represent s.e.m.).

To assess to what extent the H_2_S vasodilatory effects are related to reduction of endogenous NO to HNO and activation of the HNO–TRPA1–CGRP pathway, we first performed laser Doppler recordings of meningeal blood flow in the rat. Topical application of H_2_S induced a clear increase in blood flow (Fig. 7a,b). This effect was significantly inhibited by topical application of HC030031 as well as by intravenous (i.v.) injection of the NOS inhibitor L-NMMA (Fig. 7a,b). Some H_2_S response was retained even 90 min after L-NMMA injection, and this was completely inhibited by topical application of glibenclamide (Fig. 7a,b), the K_ATP_ channel antagonist, confirming the additional role of K_ATP_ channel activation in the action of H_2_S (refs 8, 38).

I.v. injection of 0.9 μg kg^−1^ Na_2_S led to an increase of blood flow in the rat medullary brainstem, which was followed by an increase of CGRP levels in the cerebrospinal fluid (Fig. 7c,d). CGRP release was completely abolished in the presence of L-NAME and TRPA1 antagonists (Fig. 7c,d) confirming that H_2_S has to react with NO to stimulate the TRPA1–CGRP pathway.

To strengthen our hypothesis that the observed effects of H_2_S could originate from the reaction with endogenous NO, we applied H_2_S on cultured DRG neurons loaded with the specific NO fluorescent indicator DAF–FM–DA. Cells that were exposed to 100 μM H_2_S showed lower intensity of fluorescence, implying that by H_2_S treatment NO is depleted from the cells (Supplementary Fig. 11a). This effect was not restricted to DRG neurons, but also evident in aorta rings, where the lower fluorescence intensity was again observed in H_2_S treated tissue (Supplementary Fig. 11b).

**Figure 8:**
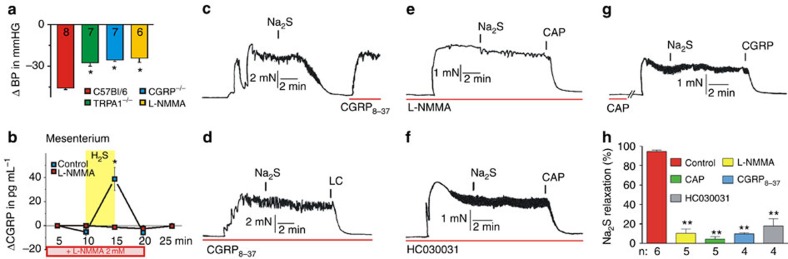
**Vasodilation caused by H**_**2**_**S depends on NO to activate HNO–TRPA1–CGRP pathway.** (**a**) Effect of H_2_S (39 μmol kg^−1^ Na_2_S) on MAP of anaesthetized mice: H_2_S-induced drop in blood pressure was reduced in TRPA1^−/−^ and CGRP^−/−^ mice and by L-NMMA (10 mg L^−1^) in drinking water for 7 days (ANOVA honestly significant difference *post hoc* test; *P<0.01; *n*=6). (**b**) Changes in CGRP levels released from the isolated mouse mesentery upon stimulation with 200 μM H_2_S following 45 min pre-treatment with 2 mM L-NMMA (*U*-test; *P*<0.004; *n*=6; all error bars represent s.e.m.). (**c**–**h**) Isometric tension recordings of phenylephrine (3 μM)-preconstricted ring segments of rat second-order mesenteric artery branches: (**c**) Na_2_S (10 μM)-induced vasodilation was reversed by CGRP_8–37_ (3 μM) and (**d**) abolished by CGRP receptor block (CGRP_8–37_). (**e**) L-NMMA (30 min; 1 mM) and (**f**) HC030031 (100 μM) pre-treatment inhibited vasodilation induced by Na_2_S. (**g**) CGRP depletion (10 μM capsaicin (CAP) prior the constriction) also abolished the Na_2_S-induced blood vessel relaxation. Capsaicin (10 μM), and the smooth muscle relaxants CGRP (10 nM) and KATP opener levcromakalim (LC, 1 μM) were added at the end to confirm intact vasodilator properties. (**h**) Percentage of Na_2_S-induced relaxation in all these experiments is expressed as mean±s.e.m. (ANOVA, Bonferroni *post hoc* test; **P<0.01; *n* given in the figure).

In addition to local vasodilation, the effect of H_2_S on arterial blood pressure was monitored. We first assessed whether the TRPA1–CGRP part of the pathway is important for the regulation of systemic blood pressure. Application of HC030031 and CGRP_8–37_ led to a rise of blood pressure in rats, similar to that observed with L-NAME application (Supplementary Fig. 11c), suggesting that both CGRP release and TRPA1 activation play a constitutive role in the regulation of blood pressure. I.v. injection of H_2_S caused a transient drop of blood pressure by 45±1 mm Hg in wild-type mice, in a similar manner and comparable amplitude as reported previously^10^. However, significant reductions of the H_2_S-induced blood pressure decrease were observed in TRPA1^−/−^ mice (by 28 mm Hg) and CGRP^−/−^ mice (by 25 mm Hg), as well as in wild types receiving L-NMMA for 7 days through the drinking water (by 24 mm Hg, Fig. 8a).

If our hypotheses were valid, part of the NOS-generated NO would react with H_2_S to give HNO and activate the TRPA1–CGRP pathway. Inhibition of H_2_S production should thus affect the MAP changes induced by NOS inhibitors. Indeed, the administration of L-NMMA led to an increase of blood pressure (12.5±1.2 mm Hg), but this change was significantly inhibited by pre-administration of H_2_S inhibitors (6.5±0.5 mm Hg) confirming that part of the vasodilation induced by NOS activity is H_2_S dependent (Supplementary Fig. 12c).

We further addressed the above question using the rat isolated mesenteric artery, which is densely innervated with CGRP-containing sensory nerve fibres and potently relaxed by exogenous CGRP as well as TRPA1 agonists^20^,^39^,^40^. The treatment with H_2_S of mouse mesenteric vessels, in which TRPA1 activators cause CGRP-mediated relaxation^40^, led to a robust CGRP release, an effect that was completely abolished by pretreatment with L-NMMA (Fig. 8b). Furthermore, using only 10 μM H_2_S, we induced almost complete relaxation of the preconstricted rat blood vessels, an effect that was completely reversed by subsequent addition of the CGRP receptor antagonist (Fig. 8c). Pretreatment with the CGRP receptor antagonist or the TRPA1 channel blocker inhibited the vasodilatory effect of H_2_S, as did pretreatment with capsaicin, which served to deplete the neurogenic pools of CGRP (Fig. 8d,f,g). Most importantly, treatment with L-NMMA completely blocked the relaxant effect of H_2_S (Fig. 8e). L-NMMA treatment had no effect, however, on ring segments of rat isolated thoracic aortas, which display a minor CGRP innervation and negligible CGRP vasodilator responses^39^, and an effect of H_2_S was observed only at toxic concentrations higher than 1 mM (not shown). This strongly suggests a systemic relevance of the interaction between H_2_S and endogenous NO to activate the HNO–TRPA1–CGRP pathway and thus contributes to the control of vascular tone.

**Figure 9:**
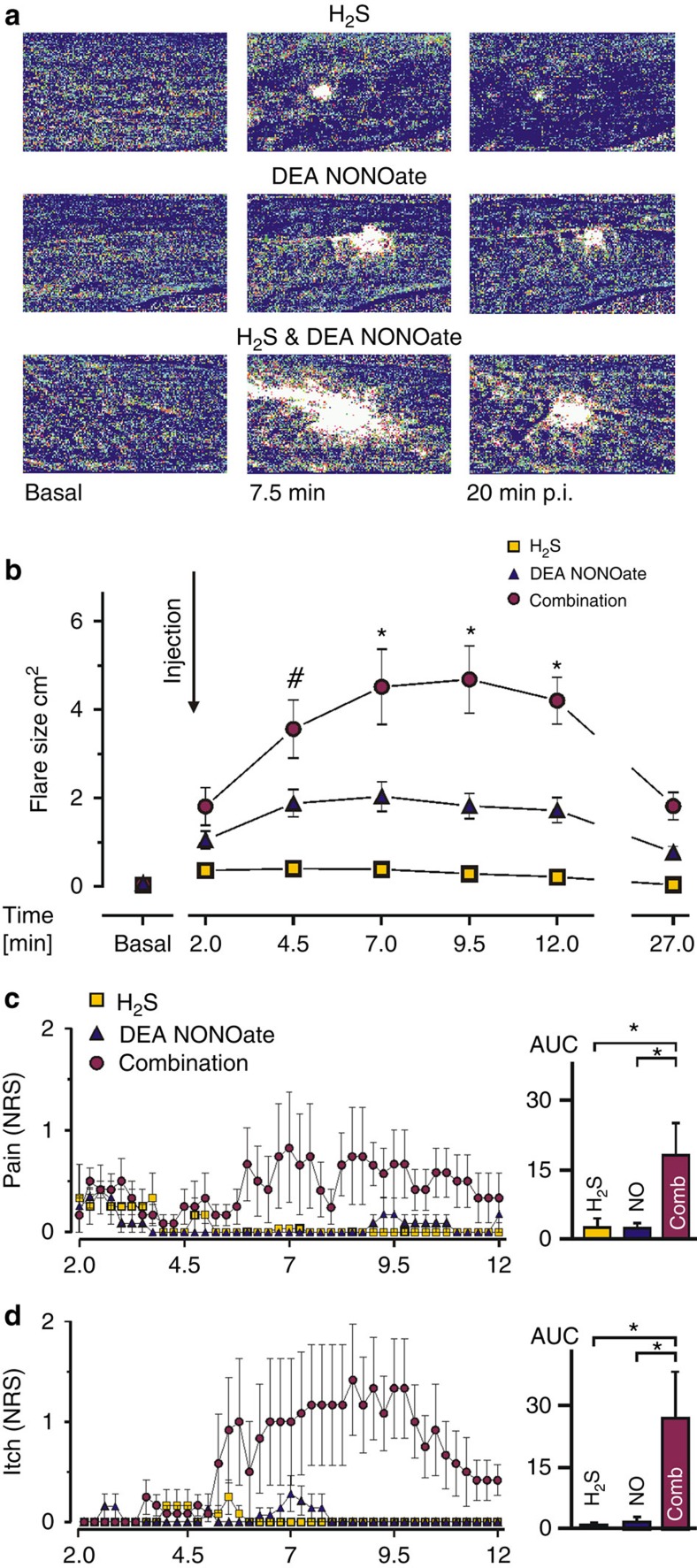
**Combination of NO and H**_**2**_**S induces axon-reflex erythema in humans.** (**a**) Laser Doppler scanned pseudocolor images of volar forearm of human volunteers (3 male, 3 female) after intracutaneous injection of DEA NONOate (0.23 μmol), Na_2_S (0.35 μmol) or both. (**b**) Flare size after intracutaneous injection of Na_2_S, DEA NONOate or combination of both. When H_2_S and NO were intracutaneously injected to the volar forearm of human volunteers, blood flow increased locally following DEA NONOate (0.23 μmol), while H_2_S (0.35 μmol) hardly evoked any response apart from slight irritation due to the injection needle. Injection of H_2_S and NO in combination however led to widespread vasodilatation in agreement with formation of an axon-reflex erythema due to activation of nerve fibers, antidromically propagated action potentials and concomitant CGRP release (repeated measures analysis of variance (ANOVA) honestly significant difference (HSD) *post hoc* tests; ^#^*P*<0.01 H_2_S and NO versus H_2_S; *P<0.01 H_2_S and NO versus H_2_S and NO; *n*=6; all error bars represent s.e.m.). (**c**) Pain and (**d**) itch ratings from the experiments shown in **a** (repeated measures ANOVA HSD *post hoc* tests; ^#^*P*<0.01 H_2_S and NO versus H_2_S; *P<0.01 H_2_S and NO versus H_2_S and NO; *n*=6; all error bars represent s.e.m.).

To add human translational evidence six adult volunteers (three male, three female) received intradermal injections of DEA NONOate, Na_2_S and the combination of both, in a double-blinded study. NO induced a small, circumscribed vasodilation, suggestive for a localized activation of the classical cGMP pathway (Fig. 9a,b). H_2_S induced a minute and transient spot of increased blood flow, due to the very fast metabolic rate of its removal^41^. However, when combined, these two gasotransmitters induced a strong vasodilation with a large axon-reflex erythema characteristic for the antidromic action potential propagation into collaterals of wide-branching C-fibres that release CGRP^7^,^8^ (Fig. 9a,b; Supplementary Fig. 12e). This was accompanied by pain (Fig. 9c) and/or itch sensations (Fig. 9d) in all subjects. The pain ratings were lower than observed with AS, but alternated with itch sensations that were not previously reported with AS.

## Discussion

In the past decade both chemical and physiological research on nitroxyl (HNO) has shown that this congener of nitric oxide has distinct ways of action^3^,^4^,^6^. Studies have reported that HNO may be a co-product of NOS activity being converted back to NO by Cu/Zn superoxide dismutase and other suitable electron acceptors^42^,^43^. It has also been reported that the NOS intermediate HO-Arg can be oxidized by catalase and hydrogen peroxide or cytochrome P450 enzymes to produce HNO^44^ suggesting that HNO can not only be produced directly by NOS but also from precursors such as HO-Arg. HNO operates together with NO to mediate the classical EDRF-induced dilatation in conduit arteries^45^,^46^,^47^ and also in mediating nitrergic neurotransmission^48^. Still, the main pathways for physiological generation of HNO and its way of action remained elusive.

Here we provide translational evidence for a direct, HNO-induced activation of neuronal TRPA1 channels, which is followed by Ca^2+^ influx and, consequently, by the release of CGRP from ubiquitous, polymodal, sensory nerve fibres and endings. These effects are completely absent in mice lacking TRPA1, blocked by a selective TRPA1 inhibitor, and not induced by a pure NO donor, leading to the conclusion that the chemosensory TRPA1 channel is the prime target for HNO-induced CGRP release and resulting cardiovascular effects.

Recent emergence of another gasotransmitter, H_2_S, raised the possibility for its interaction with NO. Several groups have considered that H_2_S effects could be linked to NO, including the early studies that showed physiological effects of H_2_S (ref. 13). In addition, positive inotropic effects of these combined gasotransmitters on the heart have been demonstrated^15^,^49^, which is one of the hallmarks of HNO physiology. However, these studies mainly used sodium nitroprusside (SNP) as a source of NO. Although it has ‘NO-like’ physiological effects, such as cGMP-dependent vasodilation, SNP does not release NO unless exposed to light^50^. We have recently published that the above mentioned observations were in fact artefacts due to the fact that SNP reacts with H_2_S directly to generate HNO and thiocyanates, without the actual involvement of NO^50^.

Some of us were the first to identify that H_2_S could be involved in TRPA1 receptor activation^19^ with several follow-up studies suggesting the same^51^,^52^. Furthermore, previous studies have also linked H_2_S to TRPA1 and microvascular blood flow mediated by CGRP^53^ and H_2_S and TRPA1/TRPV1 intestinal pro-secretory actions^54^. However, only very high concentrations of H_2_S activated TRPA1 (as we also observed, Supplementary Fig. 10). These effects are, however, most likely outside the physiological concentration range and should stand for inhibition of cell respiration^33^ and/or generation of ROS^34^ both of which can induce TRPA1 activation^35^,^36^. In addition, polysulphides, inevitable contaminants of NaHS solutions used in these studies^19^,^51^,^52^, could be the reason for the activation of the TRPA1, as shown recently^55^. H_2_S, just like NO, cannot react directly with thiols due to thermodynamic constraints^37^.

Using selective methods for *in vitro* and intracellular HNO detection, our data suggest that the gasotransmitter H_2_S may transform endogenous NO to HNO, which activates the HNO–TRPA1–CGRP cascade, suggesting broad physiological relevance of the findings. Mechanistic details of direct reaction between NO and H_2_S will be subject of further studies. As we previously demonstrated, it is plausible that H_2_S-mediated generation of HNO *in vivo* could be additionally related to its reaction with *S*-nitrosothiols^16^, as well as with metal nitrosyls^56^.

Data supporting the notion that H_2_S may react with NO to give HNO, and our demonstration that H_2_S effects could be diminished by either blocking NOS activity or deleting TRPA1 or CGRP are in favour of a new signalling pathway for cardiovascular control. In addition, co-expression of TRPA1 and CBS in small to medium-sized sensory neurons and axons together with the recent demonstration of co-expression of TRPA1 and nNOS^32^ suggest a structural and functional organization for constitutive HNO generation and subsequent activation of TRPA1-dependent CGRP release. This functional unit is of importance in the regulation of peripheral blood flow (as demonstrated in dura mater and brainstem) and even of systemic blood pressure. In addition, positive inotropic and lusitropic cardiac effects of circulating and/or paracrine CGRP have been reported^3^,^4^,^7^. The positive inotropic and lusitropic effects of HNO were originally ascribed to CGPR release and completely blocked by the use of CGRP receptor antagonist, CGRP_8–37_ (ref. 4). Expression of CRLR in cardiomyocytes was previously documented for the rat heart^57^ and we confirmed its presence at the protein level in human hearts (both, the membranous and cytoplasmic distribution) using indirect immunofluorescence. These results explain the CGRP receptor-mediated positive inotropic effects that have previously been reported from isolated trabecular muscles of the human heart^58^. Also on rat cardiomyocytes, direct CGRP-mediated inotropic and lusitropic effects have recently been demonstrated^59^. Thus, the failing heart may gain particular benefit from CGRP release, as coronary blood flow is increased and afterload (peripheral resistance) reduced by CGRP^60^. Thereby, it would not really matter whether beneficial effects derive from circulating plasma levels of CGRP or from paracrine release from ubiquitous sensory nerves that accompany every peripheral blood vessel.

**Figure 10:**
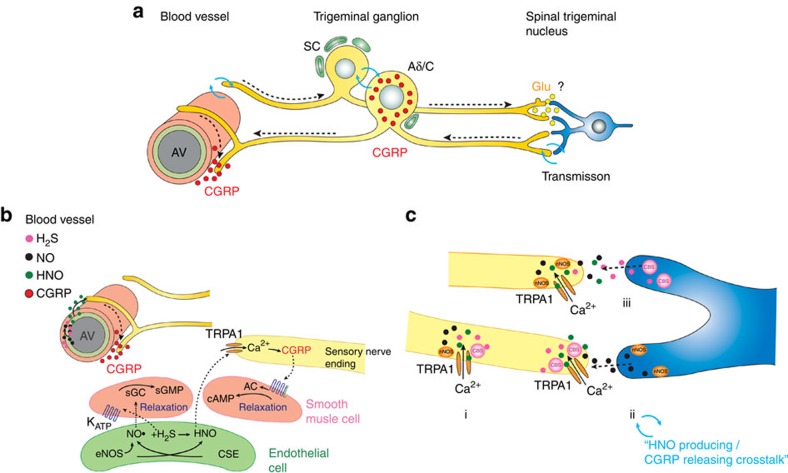
**H**_**2**_**S-NO-HNO–TRPA1–CGRP pathway in neurovascular regulation and synaptic transmission.** (**a**) The trigeminal system as an example of CGRP-containing nerve fibres and its neuronal and vascular interaction sites. By means of diffusion of H_2_S or NO, produced either in the endothelium or in neurons, there are several possibilities of their reaction leading to formation of HNO that targets the cysteines of TRPA1 in close vincinity. (**b**) TRPA1/CGRP expressing nerve endings in the periphery communicate with the smooth muscle cells surrounding the endothelium of blood vessels. Endothelial cells are known to produce NO and H_2_S, both of which freely diffuse and activate guanylyl cyclase and K_ATP_ channels, respectively, to induce vasodilatation. However, H_2_S and NO also react with each other to give HNO, which could reach paravascular TRPA1-expressing sensory nerve fibres, inducing Ca^2+^ influx and CGRP release. (**c**) Other potential sites of NO–H_2_S interaction in neurons: (i) TRPA1 channels are co-expressed with nNOS and CBS in primary afferents forming a functional signalling complex that leads to confined HNO generation and TRPA1 gating upon activation of the gasotransmitter-generating enzymes. In addition, NO (ii) or H_2_S (iii) could originate from either side of a synaptic cleft (or from nearby axons of passage) and freely diffuse into adjacent neurons (or nerve fibres). There, they react with their counterpart producing HNO in vicinity of TRPA1, which leads to its activation, Ca^2+^ influx and release of CGRP. Apart from its vascular functions, CGRP acts as a co-transmitter, facilitating synaptic transmission, which may play a role in migraine headaches. Glu, glutamate; SC, satellite cells.

All three, NO, H_2_S and HNO are freely diffusible, so several possibilities could be envisioned for TRPA1 activation and CGRP release (Fig. 10): (i) NO and H_2_S produced in endothelium react to give HNO that could diffuse and activate nearby nerve endings expressing TRPA1 and releasing CGRP that relaxes vascular smooth muscles; (ii) production of H_2_S and NO from colocalized CBS and neuronal NOS leads to intracellular HNO formation and TRPA1 activation; (iii) taking into account that constitutive levels of NO in neurons are very low^61^ it is also plausible that, for instance in the CNS, astrocyte-derived NO, as a paracrine signal, meets with endothelial or neuronal H_2_S, forming HNO, which activates TRPA1 in primary afferent peptidergic terminals. Recent work showed that the NO vasodilatory effect on aorta rings is partially blocked by inhibiting H_2_S production, *vice versa* H_2_S effects are diminished by inhibiting NO production, further strengthening the link between these gasotransmitters^14^. Our data on isolated blood vessels suggest that most of H_2_S-induced vasodilation is directly dependent on its reaction with NO to form HNO, as well as on functional presence of TRPA1 and CGRP.

Formation of disulphides as a mode of channel activation, as shown in this study, may have a broader impact on understanding the multiple mechanisms for TRPA1 activation, as other activators could use the same mechanism. We^22^ and others^62^ have recently demonstrated the formation of disulphides in the N terminus of TRPA1 by other endogenous (methylglyoxal) and exogenous (*N*-methylmaleimide) activators. Although the initial reaction with C621, C641 and/or C665 is unquestionable, the extent to which neighbouring cysteines interact, as well as the extent to which the size, hydrophilicity and charge of the activator could determine the half-life of the disulphide-induced conformational change, remain to be elucidated in further studies. In case of HNO, the activation is particularly long lasting and could be reversed by a reducing agent. Thus, if reducing equivalents are scarce, as in conditions of oxidative stress, and/or when H_2_S and NO are produced in excess, pathophysiological effects of the HNO–TRPA1–CGRP (plus substance P) pathway are well conceivable. Being a nociceptive transduction channel in the first place, TRPA1 contributes to pain/itch sensations and possibly excessive CGRP release into the jugular venous blood^63^, for example, where also plenty of metabolic NO products are found during migraine attacks^64^.

The deciphering of the HNO–TRPA1–CGRP pathway provides several new targets for future drug design. A century after the demonstration that the classical TRPA1 agonist mustard oil acts as a strong vasodilator^65^, the ion channel responsible for this action could be considered as a co-mediator of endogenous ligand effects that lower the blood pressure and strengthen the heart^66^,^67^ both effects of CGRP that would be highly appreciated in the treatment of cardiac failure.

## Methods

### Chemicals

Stock solutions of DEA NONOate and AS were prepared in 10 mM KOH and diluted with physiological buffer (pH 7.4) immediately before use to minimize the loss of activity due to spontaneous decomposition of these salts at neutral pH. 300 mM phosphate buffer (KPi) was prepared with nanopure water, stirred with Chelex-100 resins to remove traces of heavy metals (and when appropriate supplemented with 100 μM neocuproine) and kept above the resins until used. Sodium sulphide (Na_2_S) was purchased as anhydrous, opened and stored in a glove box (<1 p.p.m. O_2_ and <1 p.p.m. H_2_O). 100 mM and 10 mM stock solutions of sodium sulphide were prepared in the glove box using argon-bubbled nanopure water and stored in glass vials with PTFE septa at +4 °C, for a maximum of one week. The concentration of H_2_S was determined using a H_2_S selective electrode (World Precision Instruments, USA). Gas-tight Hamilton syringes were used for handling Na_2_S solution.

### Cell culture

DRGs of C57Bl/6 or TRPA1^−/−^ mice were excised and transferred into Dulbecco’s modified Eagle’s medium containing 50 μg ml^−1^ gentamicin. Following treatment with 1 mg ml^−1^ collagenase and 0.1 mg ml^−1^ protease for 30 min ganglia were dissociated by trituration with a fire-polished silicone-coated Pasteur pipette. For plating, coverslips were coated with poly-D-lysine (200 μg ml^−1^, Sigma-Aldrich, Germany) and cells were cultured in TNB 100 cell culture medium supplemented with TNB 100 lipid–protein complex, 100 μg ml^−1^ streptomycin and penicillin (all from Biochrom, Berlin, Germany) and mouse NGF (100 ng ml^−1^, Alomone Labs, Tel Aviv, Israel) at 37°C and 5% CO_2_. All experiments were performed within 24 h of dissociation.

HEK 293t cells were transfected with plasmids of hTRPV1, hTRPA1, mTRPA1 or mutant hTRPA1 (1 μg each) using Nanofectin (PAA, Pasching, Austria). Twenty-four hours after transfection cells were plated on coated coverslips and used for calcium imaging experiments the same day. Human and mouse TRPA1 cDNA and cDNA of a mutant hTRPA1 lacking lysine and/or cysteine residues in the intracellular domain (C621S, C641S, C665S +/− K710R) were a kind gift from Dr Sven-Eric Jordt (Department of Pharmacology, Yale University, New Haven, USA).

Chinese hamster ovary cells expressing mTRPA1 were a gift from Dr Ardem Patapoutian (The Scripps Research Institute, La Jolla, USA).

### Animals

TRPA1 knockout mice were a gift from Drs. David Corey and Kelvin Kwan (Harvard University, Boston, USA); TRPV1^+/−^ came from Dr John Davis (formerly GSK, Harlow, UK) and CGRP (Calca)^+/−^ from Dr Jean-Pierre Changeux (Institut Pasteur, Paris, France). For measuring CGRP release and culturing neurons of dorsal root ganglia (DRG), mice and rats were suffocated in rising CO_2_ atmosphere. For *in vitro* studies, animals of both sexes were used. Rats weighed up to 400 g and mice up to 28 g. Euthanasia as well as *in vivo* studies on isoflurane anaesthetized mice and rats were performed in accordance with ethical principles for animal care and treatment of the International Association for the Study of Pain. All experimental procedures were carried out in compliance with the guidelines for the welfare of experimental animals stipulated by the Federal Republic of Germany and approved by the animal protection authorities of the Friedrich-Alexander University Erlangen-Nuremberg and the local district government (reference number 54-2532.1-21/12). TRPA1 and CGRP knockout mice were backcrossed over more than nine generations to C57Bl/6J background and constantly genotyped.

### Human tissue samples

The retrospective examination of pseudonymized human (cardiac) tissue samples (from archival tissues or obtained from autopsies with permission by relatives and institutional approval for collection of research biomaterials), was performed in compliance with university ethics committee guidelines and the German federal law for correct usage of archival tissue for clinical research (Reference Dtsch. Arzteblatt 2003:100: A1632).

### Statistics

Comparison of two groups was performed by the respective *t*-test and with a non-parametric test for *n*<10. More than two groups were compared by analysis of variance following least significant difference or honestly significant difference *post hoc* tests as stated using Statistica 7 software (StatSoft, Tulsa, USA). Differences were considered significant at *P<0.05.

### HNO fluorescent imaging

For nitroxyl fluorescent imaging, DRG neurons were plated in poly-D-lysine coated 35 mm high μ-dishes ibiTreat (Ibidi, Germany). Cells were incubated with 10 μM CuBOT1 in Hank’s buffer for 20 min. After washout, cells were treated with AS, DEA NONOate, Na_2_S (100 μM each) or medium as a control for 15 min. In addition, cells were preincubated with 1 mM L-NMMA, 1 mM oxamic acid or combination of both for 2 h or kept in medium without arginine, cysteine or without both for partial depletion of NO and/or H_2_S, before staining with CuBOT1, to quantify the effects on basal HNO production. Fluorescence microscopy was carried out using Carl Zeiss Axiovert 40 CLF inverted microscope, equipped with green fluorescent filters and AxioCam ICm1. Images were post processed in ImageJ software^68^ where semi-quantitative fluorescence intensity was determined.

### Intracellular detection of NO with DAF-FM

For the intracellular detection of NO, cells (cultured DRG neurons) were incubated with 5 μM 4-amino-5-methylamino-2′,7′-difluorescein diacetate (DAF–FM–DA) for 30 min in the absence or presence of 100 μM sodium sulphide. The cells were washed three times and placed in external solution (as used for calcium imaging experiments, see below). The dye was excited with 488 nm laser line on LSM 780 confocal laser scanning system (Carl Zeiss MicroImaging, Jena, Germany) equipped with an Argon laser mounted on an inverted Axio Observer Z1. All experiments were performed in triplicate. Images were processed in ImageJ software where semi-quantitative fluorescence intensity was determined.

### Analysis of NO and H_2_S

Two microlitres of 50 mM KPi buffer pH 7.4 was added into the chamber and the electrodes were immersed into it. Depending on the type of measurement, different concentrations of Na_2_S solution were injected followed by the addition of either NO or DEA NONOate solutions. The studies were also performed with NO or DEA NONOate being injected first and then followed by injection of H_2_S. The fate of H_2_S and NO during the course of the reaction was monitored by a Free Radical Analyzer (WPI, USA) connected to a computer equipped with DataTrax software for the signal processing. Experiments were performed in a four-channel chamber (WPI) with both electrodes at the same time or each of them separately. When needed the solutions were degassed with argon and kept closed in measuring chamber securing anaerobic conditions, which were constantly monitored by additional oxygen electrode.

### Amperometric detection of HNO

Measurements of HNO concentration were carried out with our previously described method based on a three-electrode system consisting of platinum counter electrode, Ag/AgCl reference electrode and a gold working electrode modified with a monolayer of cobalt porphyrin with 1-decanethiol covalently attached. The method has been demonstrated to be specific for HNO, showing no interference or spurious signal due to the presence of NO, O_2_, NO_2_^−^ and other RNOS^29^ Signal recording was performed with a TEQ 03 potentiostat. For each case, we also confirmed that the maximal concentrations (2 mM) of NO, H_2_S and cysteine produced very small signals that can be disregarded.

### High-resolution ESI–TOF–MS

Being previously degassed with argon for 5 min, 20 μM model peptide was exposed to 1 mM H_2_S, 1 mM DEA NONOate or the combination of both. The samples were analysed on maXis, a high-resolution ESI–TOF mass spectrometer (Bruker, Bremen, Germany). The samples were injected using a syringe pump at a flow rate of 240 μl h^−1^. Nitrogen was used as the nebulizing gas at a pressure of 10 psi and as the drying gas at a temperature of 180 °C and a flow rate of 5  min^−1^. All experiments were carried out in the positive ion mode and obtained spectra deconvoluted and further processed in Data Analysis software provided by Bruker Daltonics. Analytical samples were prepared by mixing peptide samples in water with a mixture of 0.1% formic acid in water/acetonitrile (1:1, v/v).

### TRPA1 purification

For purification of mTRPA1 protein mTRPA1, DNA was cloned into a pcDNA3.1/V5–His-TOPO vector (Invitrogen, Life Technologies, Darmstadt, Germany). hTRPA1 as well as hTRPA1-C621S/C641S/C665S were cloned into the same vector and truncated to the N-terminal segment hTRPA1-1–719-V5–His. HEK 293t cells were transfected with the plasmid using Nanofectin (PAA, Pasching, Austria) and cultured for 2.5 days in 175 cm^2^ flasks before collection and purification of V5–His tagged mTRPA1 protein. The tag did not seem to influence function of TRPA1 as in Ca^2+^ imaging activation by AS and carvacrol showed comparable results to the untagged receptor channel. V5–His tagged mTRPA1-expressing HEK cells were collected in TNE buffer (0.15 M NaCl, 20 mM Tris–HCl, 1 mM EDTA pH 7.4) supplemented with 1% NP-40 and 10% proteinase inhibitor cocktail (Sigma-Aldrich) and lysed by trituration with a syringe and 26G needle. The cell extract was placed on a rotator (6 r.p.m.) at 4°C for 30 min and centrifuged. The supernatant was purified by immobilized metal affinity chromatography on a column with nickel beads following the instructions of the manufacturer (Sigma-Aldrich). The presence of TRPA1 was confirmed with anti-V5 antibodies (mouse monoclonal IgG_2a_, R961-25 Invitrogen).

### Modified biotin-switch assay for disulphide bond detection

For disulphide bond detection, purified mTRPA1 channel (50 μM) was first treated with 1 mM DTT and subsequently purified using Micro Bio-Spin Columns with Bio-Gel P-6 (Bio-Rad). The protein was then exposed to either 500 μM or 1.5 mM AS for 15 min, while a sample that was first modified with IA to block all the available SH groups before application of 1.5 mM AS was used as negative control. The samples were desalted on Micro Bio-Spin Columns with Bio-Gel P-6 (Bio-Rad) and processed as follows: (i) 5 mM IA (45 min at 37 °C), (ii) 1 mM DTT (1 h at 37 °C) and (iii) 5 mM biotin-maleimide (45 min at 37 °C). The presence of biotinylated proteins was confirmed with mouse monoclonal anti-biotin peroxidase-labelled antibodies (Clone BN-34, A0184, Sigma-Aldrich).

### MALDI-TOF characterization of AS-induced modification of hTRPA1

A model peptide of the intracellular N-terminal part of hTRPA1 including amino acids 607-670 (UniProt database, O75762, Thermo Fischer Scientific) was used to evaluate AS effects on cysteines. A quantity of 50 μM peptide in 20 mM ammonium bicarbonate buffer pH 7.4 was treated with 500 μM Angeli's salt for 15 min and then incubated with 1 mM IA for 45 min. Hydrophobic MALDI-TOF plate was pre-crystallized with sinapinic acid matrix, a supersaturated solution of 4-hydroxy-3,5-dimethoxycinnamic acid in acetonitrile, 0.1% TFA (50:50, v/v). Samples were mixed with sinapinic acid in a 1:3 ratio and spotted onto the plate.

### *Ab initio* peptide structure prediction

A model structure of the peptide containing critical cysteine residues was obtained by *ab initio* protein folding and protein structure prediction using QUARK software^69^.

### Detection of disulphides, *S*-nitrosothiols and persulphides

DRGs were prepared as described above. In addition, cells were treated with 2 mM combination of oxamic acid and L-NMMA for 12 h. After that cells were lysed in TNE buffer (0.15 M NaCl, 20 mM Tris–HCl, 1 mM EDTA pH 7.4) supplemented with 1% NP-40, 10% proteinase inhibitor cocktail (Sigma-Aldrich) and 50 mM MSBT-A^37^ using ultrasonicator. TRPA1 was immunoprecipitated with anti-TRPA1 antibodies (rabbit polyclonal IgG, ab58844, Abcam) and the samples split on three to be analysed for: (i) disulphide formation by reducing the proteins with DTT, removing DTT on Micro Bio-Spin Columns with Bio-Gel P-6 (Bio-Rad) and labelling with biotin-maleimide; (ii) *S*-nitrosothiol content, by treating the extracts with 1 mM ascorbate, 5 μM copper sulphate and biotin-maleimide; and (iii) persulphide formation, by treating the cells with CN-biotin^37^. The presence of biotinylated proteins was confirmed with mouse monoclonal anti-biotin peroxidase-labelled antibodies (Clone BN-34, A0184, Sigma-Aldrich).

### Patch clamp

Membrane currents were acquired with an EPC10 USB HEKA amplifier (HEKA Elektronik, Lamprecht, Germany), low passed at 1 kHz, and sampled at 2 kHz. Electrodes were pulled from borosilicate glass tubes (TW150F-3; World Precision Instruments, Berlin, Germany) and heat polished to give a resistance of 3–5 Mω. The standard external solution contained (in mM) NaCl 140, KCl 5, MgCl_2_ 2, EGTA 5, HEPES 10 and glucose 10 (pH 7.4 was adjusted with tetramethylammonium hydroxide). The internal solution contained (in mM) KCl 140, MgCl_2_ 2, EGTA 5 and HEPES 10 (pH 7.4 was adjusted with KOH). If not otherwise noted, cells were held at −60 mV. For IV curves with and without Angeli's salt, currents were measured during 500 ms long voltage ramps from −100 to +100 mV. All experiments were performed at room temperature. Solutions were applied with a gravity-driven PTFE/glass multi-barrel perfusion system. The PatchMaster/FitMaster software (HEKA Elektronik) was used for acquisition and off-line analysis.

### Ratiometric [Ca^2+^]_i_ measurements

Cells were stained by 3 μM fura-2-AM and 0.02% pluronic (both from Invitrogen) for about 30 min. Coverslips were mounted on an Olympus IX71 inverse microscope with a × 10 objective and constantly superfused with extracellular solution (in mM: NaCl 145, KCl 5, CaCl_2_ 1.25, MgCl_2_ 1,Glucose 10, HEPES 10) using a software controlled 7-channel gravity-driven common-outlet superfusion system. Fura-2 was excited at 340 and 380 nm by a Polychrome V monochromator. Images were exposed for 200 μs and acquired at a rate of 1 Hz with a 12–bit CCD camera (Imago Sensicam QE). Data were recorded and further analysed using TILLvisION 4.0.1.3 software (all from Till Photonics, Munich, Germany). Background fluorescence was subtracted before calculation of ratios. A 60 mM potassium (DRG neurons) or 10 μM ionomycin stimulus (HEK cells) was applied as a control at the end of each experiment. Averaged results are reported as means (±s.e.m.) of area under the curve (ΔF340 nm/F380 nm) for regions of interest adapted to the neurons.

### Recording of vasorelaxation

Arterial ring segments of the first and second-order branches of male Wistar–Hannover rats (250 g) mesenteric artery were carefully dissected and suspended between two stainless steel wires in temperature-controlled tissue baths (37 °C), containing aerated (20% O_2_ and 5% CO_2_ in N_2_) physiological salt solution (composition in mM: NaCl 119, KCl 4.6, CaCl_2_ 1.5, MgCl_2_ 1.2, NaHCO_3_ 15, NaH_2_PO_4_ 1.2 and D-glucose 6; pH 7.4), under a passive load of 1 mN mm^−1^ vessel length. One of the wires was connected to a force-displacement transducer model FT03 C (Grass Instruments; Rhode Island, USA) for isometric tension recording. The vessel segments were initially exposed to 10^−7^ to 10^−5^ M phenylephrine to estimate the maximal contractile capacity. Vasorelaxation was studied in arterial segments submaximally contracted with phenylephrine; the phenylephrine concentration was titrated in each experiment to obtain a stable contraction amounting to ~80 % of the maximal response. Vasorelaxation of phenylephrine (3 μM)-preconstricted ring segments of rat second-order mesenteric artery branches (*n*=4–6) was induced by 10 μM Na_2_S, without or with pretreatment for 30 min with 1 mM L-NMMA, 10 μM capsaicin, 3 μM CGRP_8–37_ and 100 μM HC030031 before the constriction. Capsaicin (10 μM), and the smooth muscle relaxants CGRP (10 nM) and K_ATP_ opener levcromakalim (LC, 1 μM) were added at the end to exclude nonspecific inhibition of the sensory nerve-mediated vasodilator pathway. Vasorelaxation was expressed as percentage reversal of the phenylephrine-induced contraction.

### Stimulated CGRP release

Stimulated CGRP release from dura of mice and rats and desheathed sciatic nerves of mice, as well as mouse heart and cerebrospinal fluid was studied^27^. Hemisected skull halves served as incubation chambers to expose the arachnoidal side of the dura to carbogen-gassed synthetic interstitial fluid (SIF), (consisting in (mM) 108 NaCl, 3.48 KCl, 3.5 MgSO_4_, 26 NaHCO_3_, 1.7 NaH_2_PO_4_, 1.5 CaCl_2_, 9.6 sodium gluconate, 5.5 glucose, 7.6 sucrose, pH 7.4 with or without compounds added for stimulation of CGRP release. Similarly, sciatic nerves and mesenteries were tied and passed through a series of incubations based on SIF each lasting for 5 min at 37 °C, while CGRP release was stimulated in the third to fifth incubation period. Samples were immediately stored on ice and CGRP was measured by a commercial enzyme immune assay (Bertin Pharma, Montigny, France). For studying CGRP release from isolated C57BL/6 mice were suffocated in raising CO_2_ atmosphere and the complete heart was excised by cutting the central blood vessels. The hearts were passed through a series of SIF incubations with or without test compounds added, spending 5 min in each test tube at 37 °C (for details see Supplementary Fig. 11a). Essentially the same procedures were applied to the C57BL/6 mouse mesentery of the small intestine that was isolated in one piece, measuring baseline CGRP neurosecretion and its changes upon H_2_S stimulation. If L-NMMA was employed to suppress NO synthesis, the compound was added during the equilibration period of the excised tissues and present during most of the experimental phase.

### Meningeal and medullary brainstem blood flow

Adult male Wistar rats weighing 300–400 g were anaesthetized with isoflurane. The animals were tracheotomized and artificially ventilated with oxygen-enriched room air and 2% isoflurane. The frequency of the ventilation was adjusted to maintain the end-expiratory CO_2_ at 3.5% to suppress spontaneous breathing. Systemic blood pressure was recorded with a pressure transducer connected to a catheter inserted into the right femoral artery. A catheter inserted into the right femoral vein served for systemic administration of drugs. The body temperature of the animals was monitored with a thermoprobe inserted into the rectum and was held at 37–37.5 °C with a feedback-controlled heating pad.

For measurements of meningeal and medullary brainstem blood flow, the head of the animal was fixed in a stereotaxic frame and the parietal bone was exposed on one side. A cranial window was drilled into the parietal bone to expose the cranial dura mater. The neck muscles were separated in the midline and the underlying atlanto-occipital ligament and spinal dura mater were opened to expose the medullary brainstem. Meningeal and medullary blood flow were measured with needle-type probes (tip diameter 0.8 mm) of a laser Doppler flowmeter (Moor Instruments, DRT4) positioned over a branch of the middle meningeal artery and on the medullary brainstem.

Blood flow was recorded at a sampling rate of 1 Hz and expressed in arbitrary perfusion units. Meningeal blood flow, systemic blood pressure and body temperature were recorded simultaneously and stored on a computer using MoorSoft software for Windows. The mean blood flow measured during a 3-min period before drug application was regarded as the basal flow. Changes induced in blood flow by the application of drugs were expressed as percentage of changes relative to the basal flow (mean±s.e.m.) calculated for the 5-min application period or separately for each minute of application. At the end of the experiments, the animals were killed with an overdose of i.v. thiopentone.

For analysis of corresponding liquor CGRP levels, the cerebrospinal fluid covering the exposed dorsal surface of the medullary brainstem was collected with a micropipette without touching the medulla. A quantity of 25 μl of sample was taken following i.v. injection of Na_2_S (70.2 μg kg^−1^) with or without blockers of the proposed signalling cascade when brainstem blood flow measurements were finished. All samples were immediately transferred to Eppendorf cups and diluted with EIA buffer at a volume of four times the volume of cerebrospinal fluid and then they were stored at −20 °C for later analysis by enzyme-linked immunoassay (Bertin Pharma). The CGRP concentration was calculated in pg ml^−1^, compensating the added volume of EIA buffer.

### Assessment of systemic blood pressure in mice

Male mice (weighing up to 25 g) were anaesthetized with isoflurane supplied with a mask and the right carotid arteries were catheterized to measure intraarterial blood pressure via a transducer connected to a polygraph (Hellige, Freiburg, Germany) while the substances were injected through the catheterized left jugular vein. AS was injected at a dose of 61 μg kg^−1^ freshly dissolved in saline pH 7.0, which also served as a control. In addition, anaesthetized C57Bl/6, TRPA1^−/−^ and CGRP^−/−^ mice were injected with 39 μmol kg^−1^ Na_2_S. One group of C57Bl/6 mice was also treated with 10 mg l^−1^ of L-NMMA in drinking water *ad lib* for 7 days prior the injection of Na_2_S. C57Bl/6 mice were also treated with 6 μg g^−1^ of oxamic acid and propargylglycine each, and after 10 min 6 μg g^−1^ of L-NMMA was injected. The reverse experiment was performed with first injecting the L-NMMA, and then the combination of OXA and PG. At the end of experiments, animals were killed by cervical dislocation in deep anaesthesia.

### Psychophysics

Studies on human volunteers were limited to some authors of the present study and experimental procedures were approved to fulfil the requirements of the Declaration of Helsinki by the ethics committee of the Friedrich-Alexander University of Erlangen-Nuremberg. AS (10 nmol kg^−1^), DEA NONOate (3.33 nmol kg^−1^) and decomposed AS as a control or in a second study DEA NONOate (3.33 nmol kg^−1^), Na_2_S (5 nmol kg^−1^) or a combination of both were intradermally injected in 70 μl volume to the volar forearm of the volunteer (*n*=6 each; 3 male and 3 female) in a double-blinded manner. Pain and itch were assessed on a numerical rating scale (NRS), ranging from 0 (no sensation) to 10 (maximum pain/itch imaginable) in 15 s intervals for a period of 10 min. Numerical ratings (0–10) to a 5 s lasting heat stimulus of 47 °C were compared before and after application of substances and the skin was tested for dynamic mechanical allodynia by applying gentle pressure with a cotton swab and stroking with a fine brush. A laser Doppler imager (LDI, Moore, London, UK) was used for recording changes in superficial blood flow. Two baseline scans of 0.5 mm spatial resolution were taken, following scans every 2.5 min starting right after the injections and a final one after 20 min. Area of superficial vasodilation was analysed with MLDI 3.0 software (Moore) and defined as pixels in which intensity exceeded the mean of basal values plus two standard deviations.

### Immunohistochemistry of aortas

For immunohistochemistry of aortas Wistar rats suffocated in rising CO_2_ atmosphere were immediately thoracotomized and the aorta from the ascending part to the abdominal part was dissected and placed in cold SIF (see above). Rest of the peritoneum was removed. At different levels, cross-sections of about 0.1 mm were cut with a sharp scalpel to produce aorta rings. For NO detection, aorta rings were preloaded with 25 μM DAF-FM for 60 min. Fluorescence microscopy was carried out using an inverted microscope (Axiovert 40 CLF, Carl Zeiss) with magnification × 20, equipped with green fluorescent filters and AxioCam ICm1. Images were processed using ImageJ software.

### Immunohistochemistry of trigeminal ganglia and brainstem

Adult Wistar rats were deeply anaesthetized by inhaling 4 % isoflurane and intraperitoneal injection of 0.5 ml Trapanal (thiopental, Nycomed, Konstanz, Germany). After quick thoracotomy, warm isotonic saline was perfused through the left ventricle for about 3 min followed by a solution of 4% paraformaldehyde in phosphate-buffered saline (PBS) for about 5 min. After opening of the occipital scull and cervical laminectomy, the brainstem was quickly dissected. Trigeminal ganglia were carefully excised from the skull base. Brainstem and trigeminal ganglia were placed in the fixation solution for about 1 h, washed in PBS, mounted on Tissue-Tek (Slee, Mainz, Germany), rapidly frozen in liquid nitrogen and stored in the freezer at −20°C. The ganglia were cut into series of 14 μm longitudinal sections using a cryostat (Leica, Bensheim, Germany). Sections were mounted on poly-L-lysine-coated slides (Sigma-Aldrich, Steinheim, Germany) and dried for 1 h at room temperature before immunostaining. After rinsing in PBS (0.01M, pH 7.4), the mounted sections were preincubated for 1 h at room temperature with a solution of 5% goat serum (Dianova, Hamburg, Germany) in PBS with 0.5% Triton X-100 and 1% bovine serum albumin. Sections were rinsed in PBS for another 5 min and incubated with two primary antibodies raised in rabbit directed against TRPA1 (Abcam, ab58844; 1:100) and in mouse directed against cystathionine beta synthase (Santa Cruz, sc-133154, 1:100) at room temperature overnight. Sections were washed with PBS three times for 5 min and incubated with fluorescent secondary antibodies for 1 h at room temperature: goat anti-rabbit IgG coupled to indocarbocyanine (Cy3, Dianova 111-165-144; 1:133) and anti-mouse IgG coupled to FITC (Sigma-Aldrich, St Louis, MO, 1:500) (4′,6-diamidino-2-phenylindole hydrochloride; Sigma-Aldrich, St Louis) for labelling of nuclear DNA was added at a concentration of 2 μg ml^−1^ to the secondary antibody. Sections were rinsed again three times for 5 min with PBS, coverslipped in Fluoromount G (Southern Biotech, Birmingham, AL), and stored at 4°C in the dark. To verify the specificity of the immunohistochemical reactions, stainings were controlled by omitting primary antibodies, which resulted in no immunofluorescence. Sections were examined and images of 1024 × 1024 pixels were obtained using a LSM 780 confocal laser scanning system (Carl Zeiss MicroImaging) equipped with an Argon laser (458, 488, 514 nm), a diode laser (405 nm), a DPSS-laser (561 nm) (LASOS Lasertechnik, Jena, Germany), mounted on an inverted Axio Observer Z1. The filter settings of the confocal scanner were: 514 nm excitation for Cy3 (MBS 458/561, filter 566–681 nm), 488 nm excitation for Alexa 488 (MBS 488/561/633), filter 493–543 nm) and 405 nm excitation for 4′,6-diamidino-2-phenylindole hydrochloride (MBS-405). A × 20 dry objective lens (numerical aperture 0.8) and a × 63 oil objective lens (numerical aperture 1.4) were used. Sequential scanning and appropriate pinhole settings minimized spectral bleed-through. For examination of colocalization of immunofluorescence, single optical sections at the same focus plane were taken separately and the three corresponding channels were merged into a 8-bit RGB tiff-file using confocal assistant software ZEN 2010.

### Immonohistochemistry of human heart tissue

The retrospective examination of pseudonymized human (cardiac) tissue samples (from archival tissues or obtained from autopsies with permission by relatives and institutional approval for collection of research biomaterials), was performed in compliance with Universitätsklinikum Ulm ethics committee guidelines and the German federal law for correct usage of archival tissue for clinical research (Reference Dtsch. Arzteblatt 2003:100: A1632). For immunofluorescence staining paraffin-embedded tissue was washed for 5 min three times with xylene, twice with 100% EtOH, once with 95% EtOH, once with 70% EtOH and once with PBS. Sections were boiled in Tris pH6.0 for antigen retrieval, rinsed in deionized water for 15 min and washed with PBS. Sections were blocked in 1% BSA, 0.3% Triton X-100 PBS and incubated with a 1:1,000 dilution of rabbit-anti-CRLR antibody (kindly provided by Nigel Bunnett/Eileen Grady). The specificity of the antibody has previously been determined^70^ and was additionally confirmed with preabsorption controls and omission of the primary antibody. Images were taken using an Olympus BX60 Fluorescent Microscope (Olympus, Melville, NY) with appropriate filter settings and software packages (MetaSystems, Altlussheim, Germany).

## Author contributions

M.R.F., P.R. and M.E. developed the concept, designed the study, analysed the data and wrote the paper. M.E. performed most of the experiments. M.R.F. provided substances and supervised all experiments. K.M. and M.D. planned and analyzed and K.M., M.D. and C.W. conducted *in vivo* blood flow experiments, CGRP release studies, immunohistochemistry of vessels and the trigeminovascular system. M.R.F. and J.M. conducted all biochemical studies. M.R.F., F.D., S.A.S., D.B., M.A.M. and I.I.B. designed and conducted chemical experiments. B.N. supervised and conducted human studies. M.F. provided tagged TRPA1 receptor DNA, purified TRPA1 protein and was involved in data interpretation. A.B. performed calcium imaging experiments on TRPV1. T.I.K. conducted CGRP release studies on mesenterium. A.Le. and A.La. supervised patch clamp experiments, J.d.l.R. performed patch clamp experiments. J.K.L. and K.D. performed immunohistochemistry on human heart tissue. J.J. and N.C. conducted blood pressure experiments on anaesthestized mice. E.D.H., P.M.Z. conducted vasorelaxation studies and were involved in data interpretation. All authors discussed the results and implications and commented on the manuscript at all stages.

## Additional information

**How to cite this article:** Eberhardt, M. *et al*. H_2_S and NO cooperatively regulate vascular tone by activating a neuroendocrine HNO–TRPA1–CGRP signalling pathway. *Nat. Commun.* 5:4381 doi: 10.1038/ncomms5381 (2014).

## Supplementary Material

Supplementary Information Supplementary Figures 1-12
