# Supplementary material for: H2S and NO cooperatively regulate vascular tone by activating a neuroendocrine HNO–TRPA1–CGRP signalling pathway
Source: Nat Commun. 2014 Jul 15;5:4381. doi: 10.1038/ncomms5381 (PMC4104458; doi:10.1038/ncomms5381)
Supplement: Supplementary Information — Supplementary Figures 1-12 [file ncomms5381-s1.pdf]

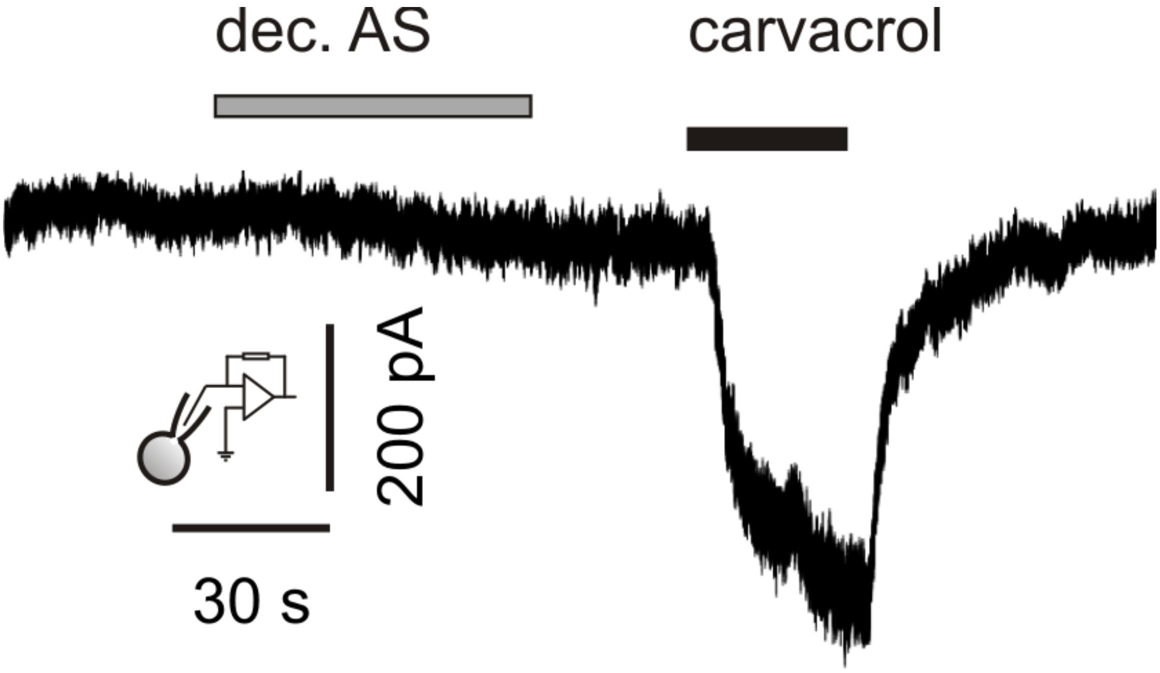

Supplementary Figure 1. TRPA1 channels are not activated when Angeli's salt is decomposed before its application

Angeli's salt (AS), commonly used as a source of HNO, decomposes in neutral buffer to give equimolar amounts of nitrite and HNO. Decomposed AS (400 µM, 60 s) did not evoke inward currents in TRPA1-expressing CHO cells responsive to carvacrol (100 µM; 30 s; n = 13).

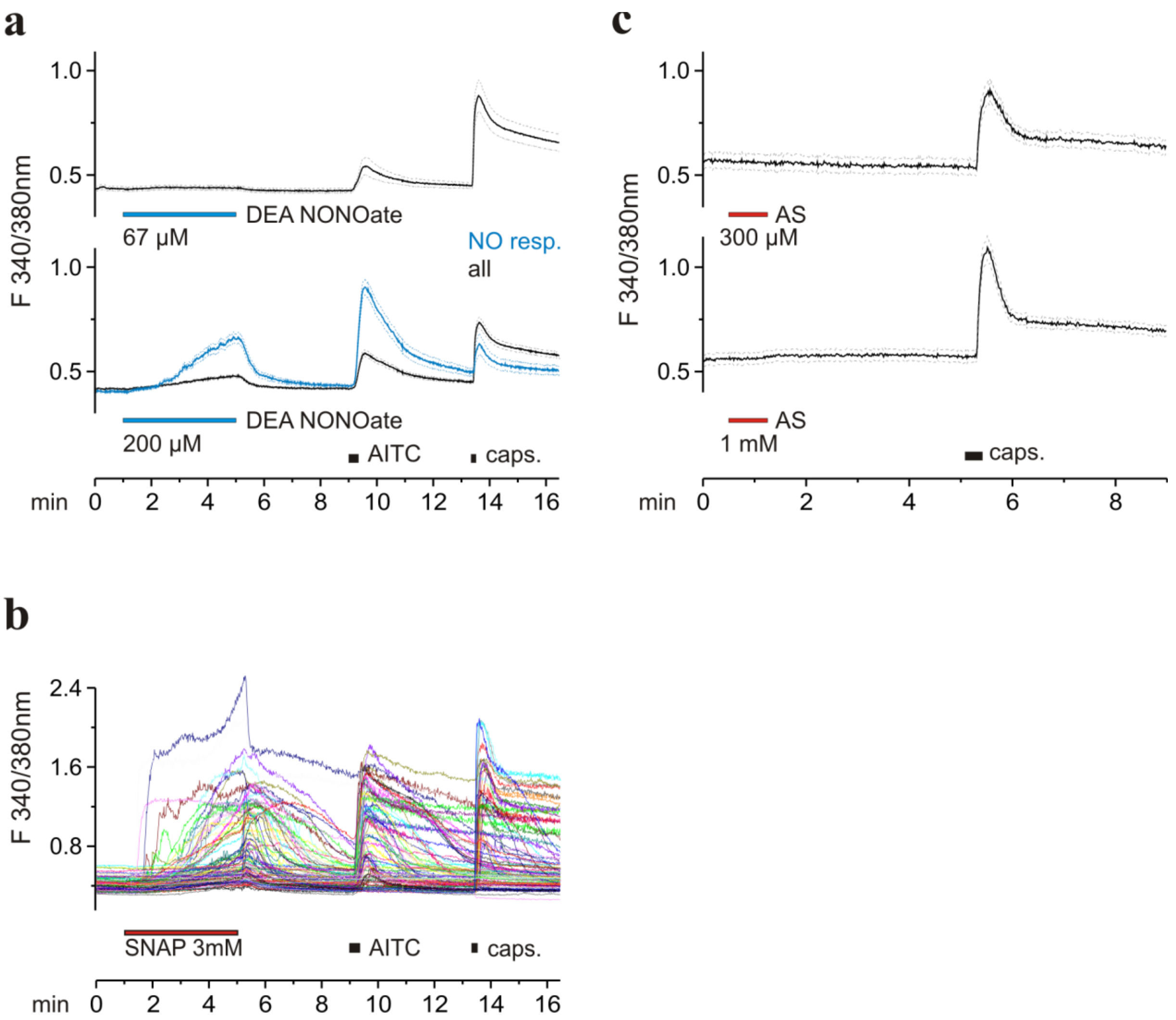

Supplementary Figure 2. NO does not activate TRPA1 and HNO does not activate TRPV1
a) DEA NONOate does not activate TRPA1 at concentrations comparable to AS. Namely, DEA NONOate decomposes to give 1.5 moles of NO per molecule with half-life similar to that of AS. Unlike HNO, however, which forms the inert gaseous molecule N_2O, NO reacts with oxygen to form more reactive molecules, NO_2 and N_2O_3. The latter acts as an *S*-nitrosating agent. **Upper panel**) 100 µM NO provided from DEA NONOate (67 µM) did not induce increases in intracellular calcium in DRG neurons even if applied for four minutes (n = 300). **Lower panel)** DEA NONOate 200 µM (a concentration providing 300 µM NO) for four minutes led to a mean increase in intracellular calcium in 19% of DRG neurons (ΔCa^{2+} > 50 nM blue, all black trace; mean ± SEM) (n = 250). **b)** In contrast to immediate responses following Angeli's salt application the *S*-nitrosating agent SNAP did only induce calcium influx in DRG neurons if applied at high concentrations (3 mM) for 4 minutes (29%, n = 228). Unlike in the AS treated cells, in SNAP treated cells Ca^{2+} levels restore to normal after the application of the activator is terminated. The use of molecules that are not real donors of NO but rather strong S-nitrosating agents (such as SNAP, SNP, *S*-nitrosocysteine or *S*-nitrosoglutathione) could lead to substantial misconceptions about the physiological mechanism behind the gained data. Our data clearly demonstrate that in a physiological range of concentrations neither NO nor *S*-nitrosothiols could activate TRPA1 channels, while this becomes possible at extremely high concentrations of NO donors or *S*-nitrosothiols. **c)** Treatment with 300 µM and 1 mM AS does not induce changes in Ca^{2+} levels in hTRPV1 expressing HEK cells. Capsaicin (0.2 µM) was used as positive control.

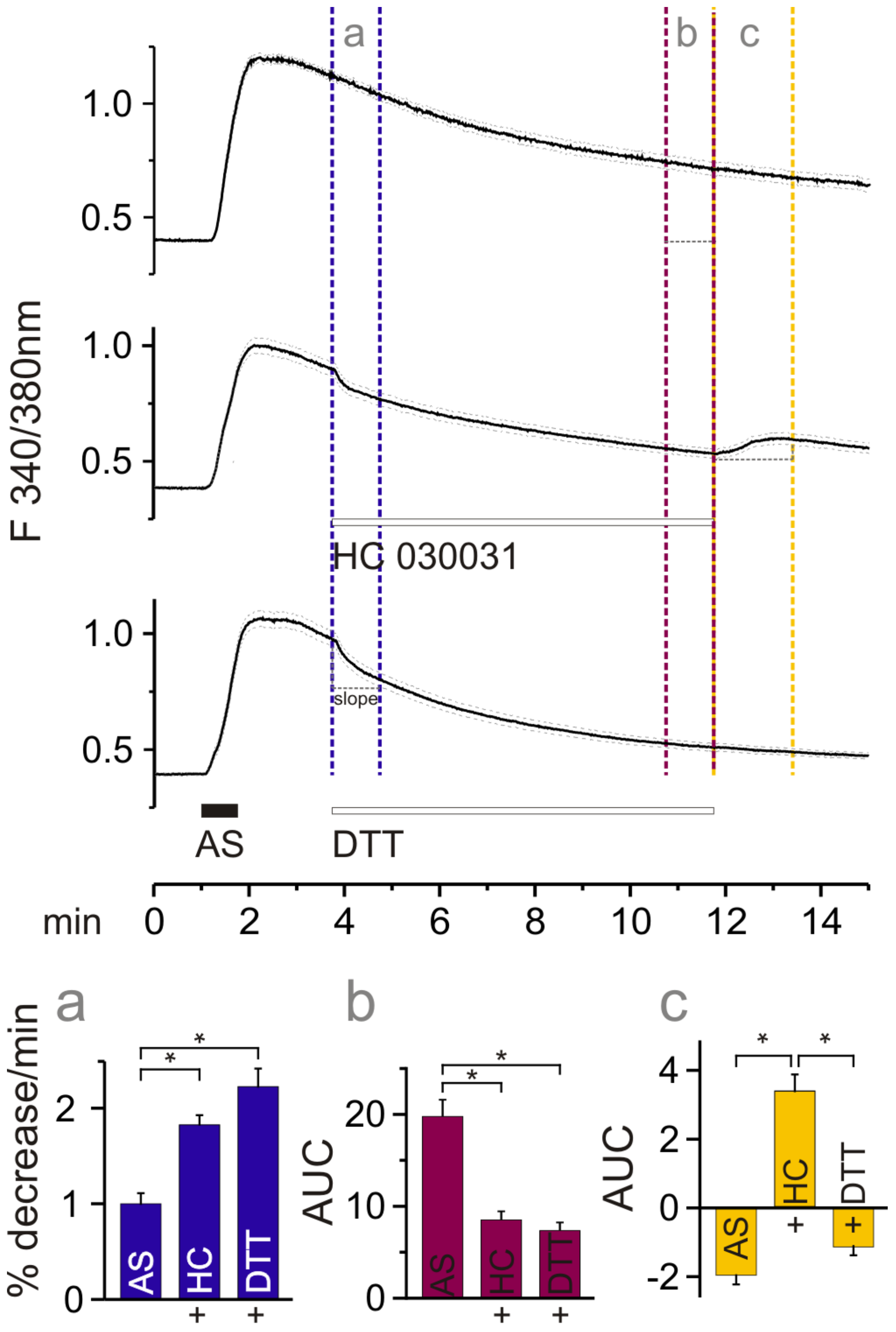

Supplementary Figure 3. HNO-induced TRPA1 activation is reversed by DTT treatment
Effects of HC030031 and dithiothreitol (DTT) on AS-induced calcium transients in DRG neurons: AS-induced (300 µM, 45 s) increases in intracellular calcium are slow in reversal (upper lane) but can be accelerated by application of HC030031 (50 µM, 10 min, middle lane) or DTT (5 mM, 10 min, lower lane) measured as the percentage of decrease within the first minute of HC/DTT application in (a). This results in a lower area under the curve of intracellular calcium calculated for the tenth minute (b) after AS application in cells treated with HC030031 or DTT. A rebound increase of intracellular calcium was observed upon washout of HC030031, but not DTT (c), suggesting that DTT deactivated TRPA1, while HC just impeded further calcium entry (all ANOVA following HSD post hoc tests; * $p < 0.001$ each; n = 67 responding DRG neurons per group; all error bars represent SEM).

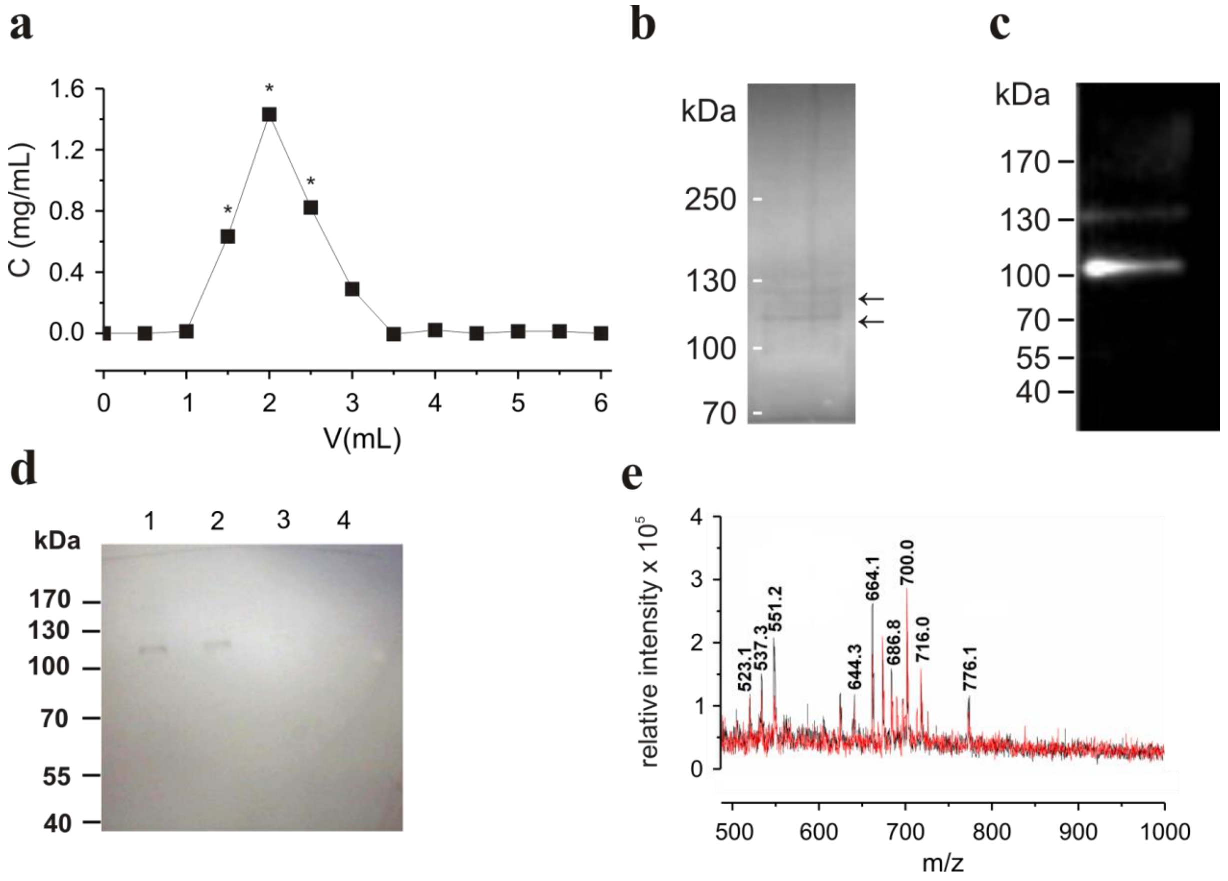

Supplementary Figure 4. HNO activates TRPA1 by modifying cysteine residues
a) Purification of mTRPA1 expressed in HEK cells. Representative chromatogram from IMAC purification step. The cell lysate was mixed with Ni-beads and incubated at 0 °C for 30 min. The column was then packed and the poly-His tagged mTRPA1 eluted with 50 mM His in 50 mM phosphate buffer pH 7.4. **b-c)** SDS-PAGE and anti-V5 immunoblot of the purified protein. **d)** Detection of disulfide bond formation on purified mTRPA1 channel protein (100 µM) treated with AS, using modified biotin-switch assay. Lane 1: TRPA1 treated with 1.5 mM AS, lane 2: TRPA1 treated with 0.5 mM AS, lane 3: untreated protein, lane 4: TRPA1 pretreated with IA and then treated with 1.5 mM AS. **e)** MALDI-TOF mass spectra of AS-treated synthetic peptide: No differences could be seen between AS-treated (red) and control (black) sample at low-mass ranges (*m/z* 450- 1000).

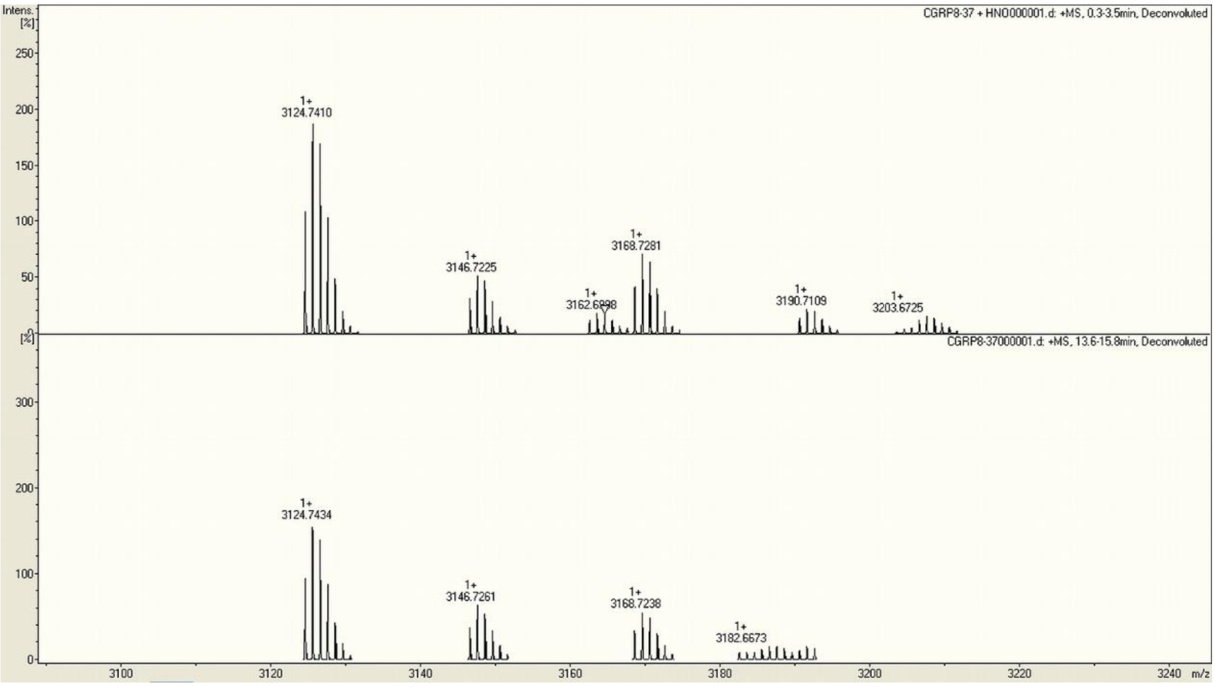

Supplementary Figure 5. Deconvoluted MS spectra of CGRP$_{8\text{-}37}$ before and after the treatment with AS

While the lower spectrum shows the peaks corresponding to the peptide in cluster with different number of Na^+ ions, the upper spectrum, corresponding to the CGRP$_{8\text{-}37}$ treated with AS shows presence of additional peaks implying that the peptide gets chemically modified, which can account for the moderate effects of this inhibitor as observed in experiments shown in **Fig. 4a**.

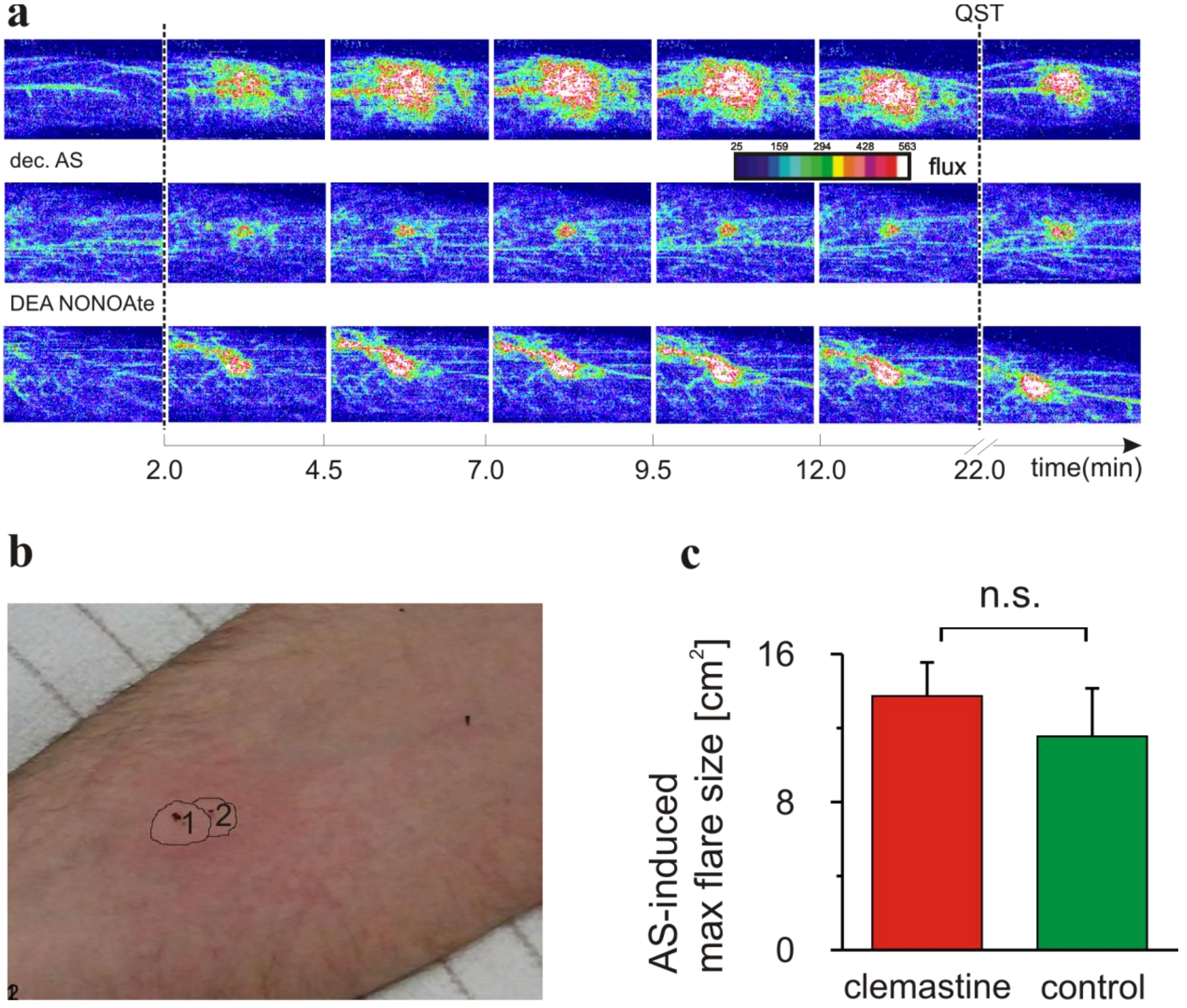

Supplementary Ffigure 6. AS-induced axon reflex erythema

a) Complete series of laser Doppler images taken before and every 2.5 minutes after double-blind intracutaneous injection of Angeli's salt (0.7 µmol), decomposed Angeli's salt or DEA NONOate (0.23 µmol) to human volunteers' volar forearms. This vasodilatation in human skin is induced by antidromic action potential conduction into collaterals of wide-branching mechano-insensitive C-nociceptors that release CGRP. DEA NONOate induced restricted, local vasodilatation which probably accounts for a direct NO/cGMP effect on the cutaneous blood vessels. Decomposed AS did neither induce a widespread axon-reflex erythema, nor a marked localized vasodilatation, but only a short-lasting weak erythema as with physiological saline injection. **b-c)** Photograph of the soft injection blebs (1-clemastine, 2-AS) and surrounding axon flare erythema and **(c)** comparison of AS-induced maximal flare size without and with pre-treatment with clemastine. 150 µL of clemastine was injected and after 10 min, 0.7 µmol AS was injected into the border of clemastine injection bleb. (repeated measures ANOVA, LSD post hoc tests; * $p \leq 0.05$; n = 6; all error bars represent SEM).

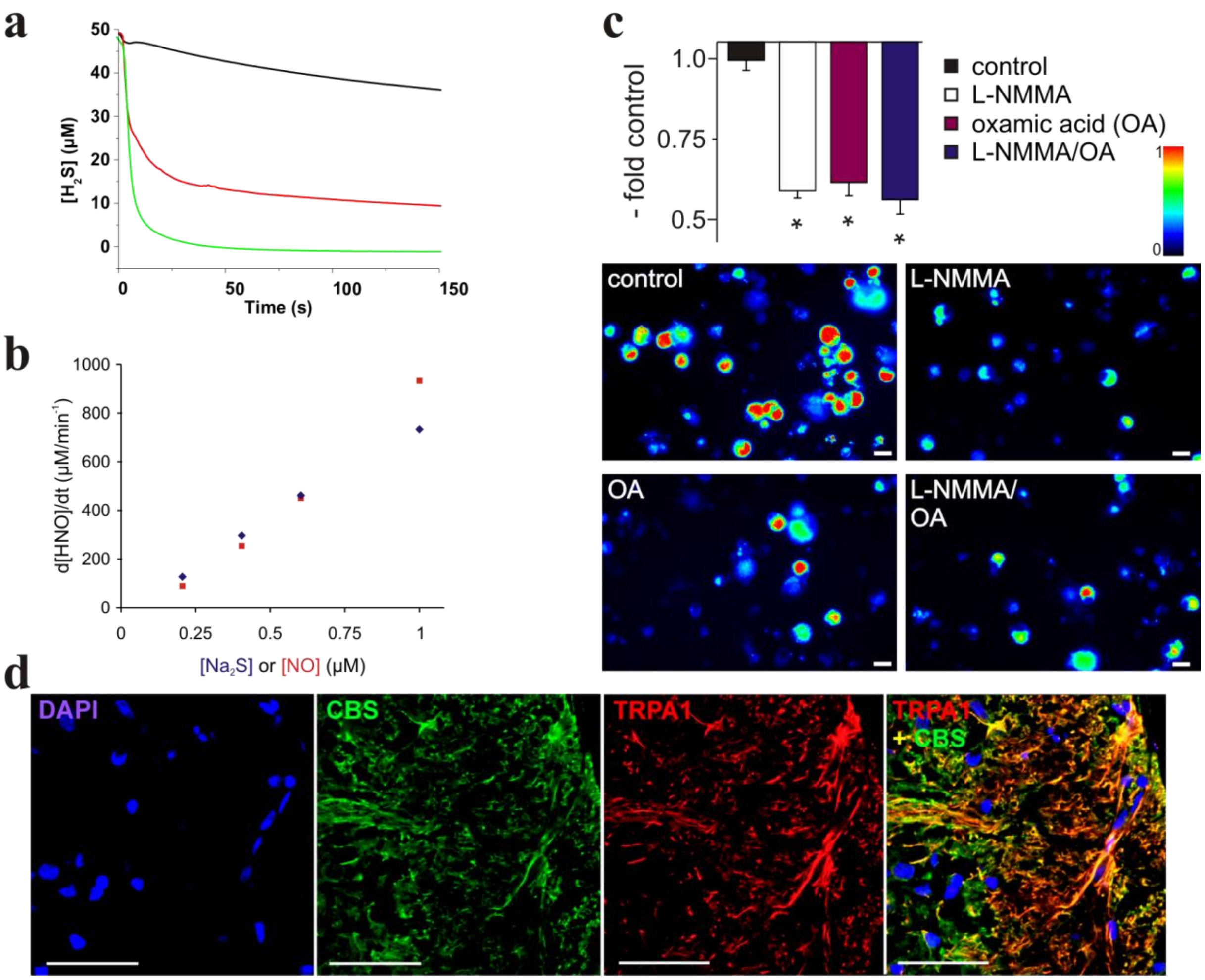

Supplementary Ffigure 7. Intracellular generation of HNO and co-localization of TRPA1 and CBS.

a) Kinetics of H_2S (50 μM) decay upon addition of NO solution (100 μM) in the presence (red) and absence (green) of 2 mM glutathione in 300 mM potassium phosphate buffer (pH 7.4). Black line represents the spontaneous decay/removal of H_2S in the same time scale. The obtained kinetic traces (red and green) suggest the same initial reaction rate in the presence and absence of glutathione. Addition of glutathione only results in a shift of a baseline. **b)** Rate of HNO formation (determined from the slope of the HNO electrode signal vs time plots) vs H_2S (blue) and NO (red) concentration, while the other reactant concentration is maintained constant and in large excess. The points represent the mean of at least three measurments. **c)** Basal fluorescence of the HNO sensor CuBOT1 in sensory neurons was reduced by inhibition of NO-synthase by L-NMMA (1 mM, 2 h of pretreatment) and cystathionine beta synthase (CBS) by oxamic acid (1 mM, 2 h of pretreatment, ANOVA HSD post hoc test; $p < 0.001$, treated vs. control respectively; $n > 150$ per group; error bars represent SEM, scale bar = 25 μm). **b)** Confocal images of a cross-section through the rat spinal trigeminal nucleus caudalis immunohistochemically double-stained with antibodies against TRPA1 (Cy3, red) and CBS (FITC, green) combined with nuclear DAPI staining (blue). Bundles of immunostained afferent nerve fibers run through the trigeminal tract (right side of images) and into the superficial laminae of the trigeminal nucleus (lamina 1 seen on left side of images). Most of the nerve fibers show the signal for TRPA1 and CBS, producing the yellow colour in the combined image. Neuronal cell bodies are not TRPA1 or CBS immunopositive (scale bar = 50 μm).

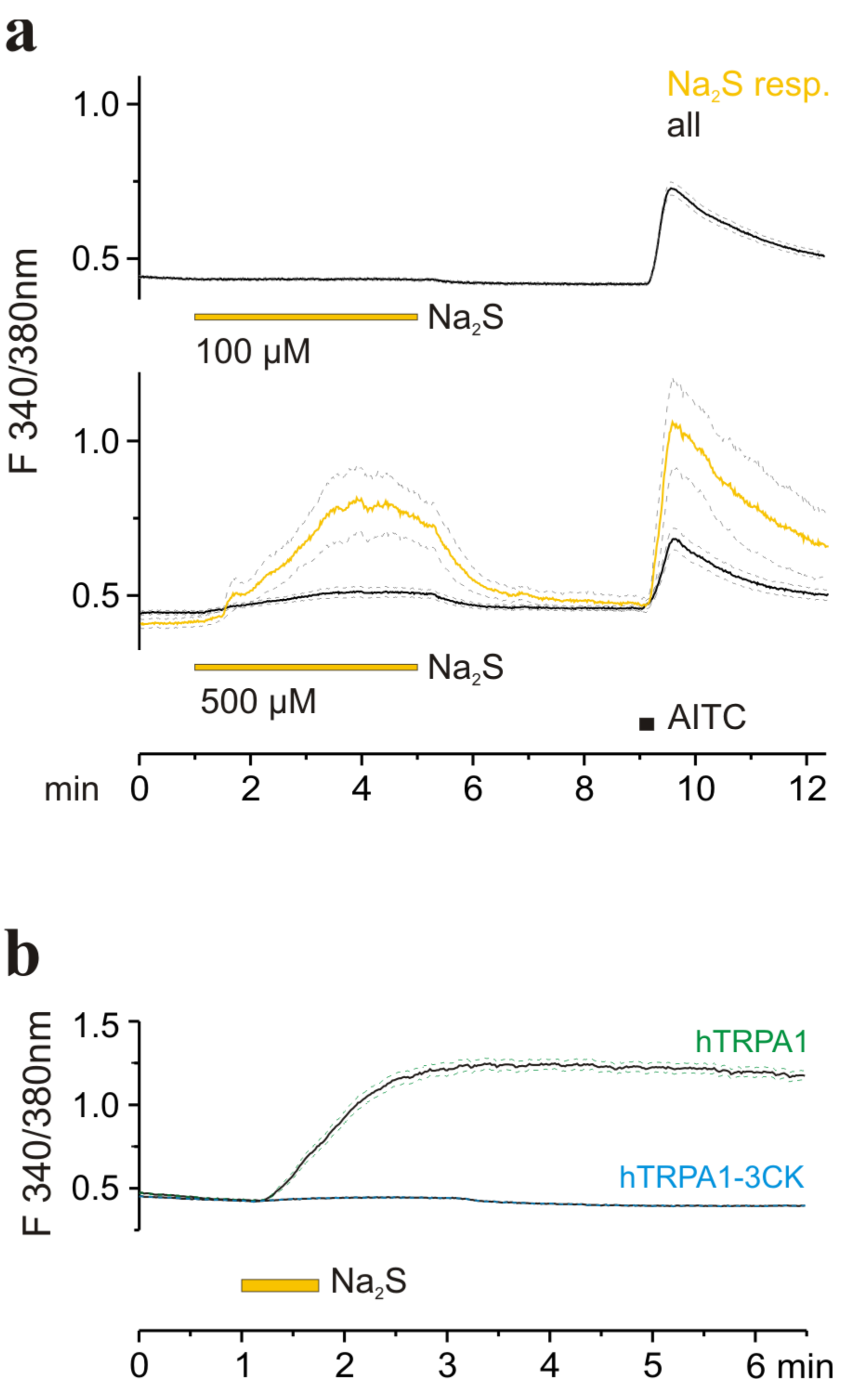

Supplementary Figure 8. H_2S alone does not activate TRPA1 in the physiological concentration range

a) Na_2S providing H_2S does not activate TRPA1 in concentrations below 500 µM in sensory neurons. **Upper panel**: 100 µM H_2S did not induce increases in intracellular calcium in DRG neurons even if applied for four minutes (n = 328), nor did H_2S at 300µM (n = 146). **Lower panel**: H_2S 500 µM for four minutes led to a mean increase in intracellular calcium in 11% of DRG neurons (yellow trace: ΔCa^{2+} > 50 nM, black trace: all cells, n = 100). **b)** However, H_2S at 1 mM applied to hTRPA1 transfected HEK cells for 120 s induced increases in intracellular calcium (25% out of 249 cells), but not in cells expressing hTRPA1-3CK, a mutant lacking essential cysteines (n = 566; mean ± SEM). Nevertheless, high concentrations of H_2S have been described to induce cytotoxicity by inhibition of cytochrome c oxidases. Inhibition of aerobic metabolism depletes ATP and causes lactic acid accumulation and furthermore block of the mitochondrial electron transport chain will increase formation of reactive oxygen species. Lactic acid and ROS have been described to activate TRPA1, the latter one by modification of critical cysteines. Therefore indirect mechanisms of TRPA1 activation by high concentrations of H_2S seem possible.

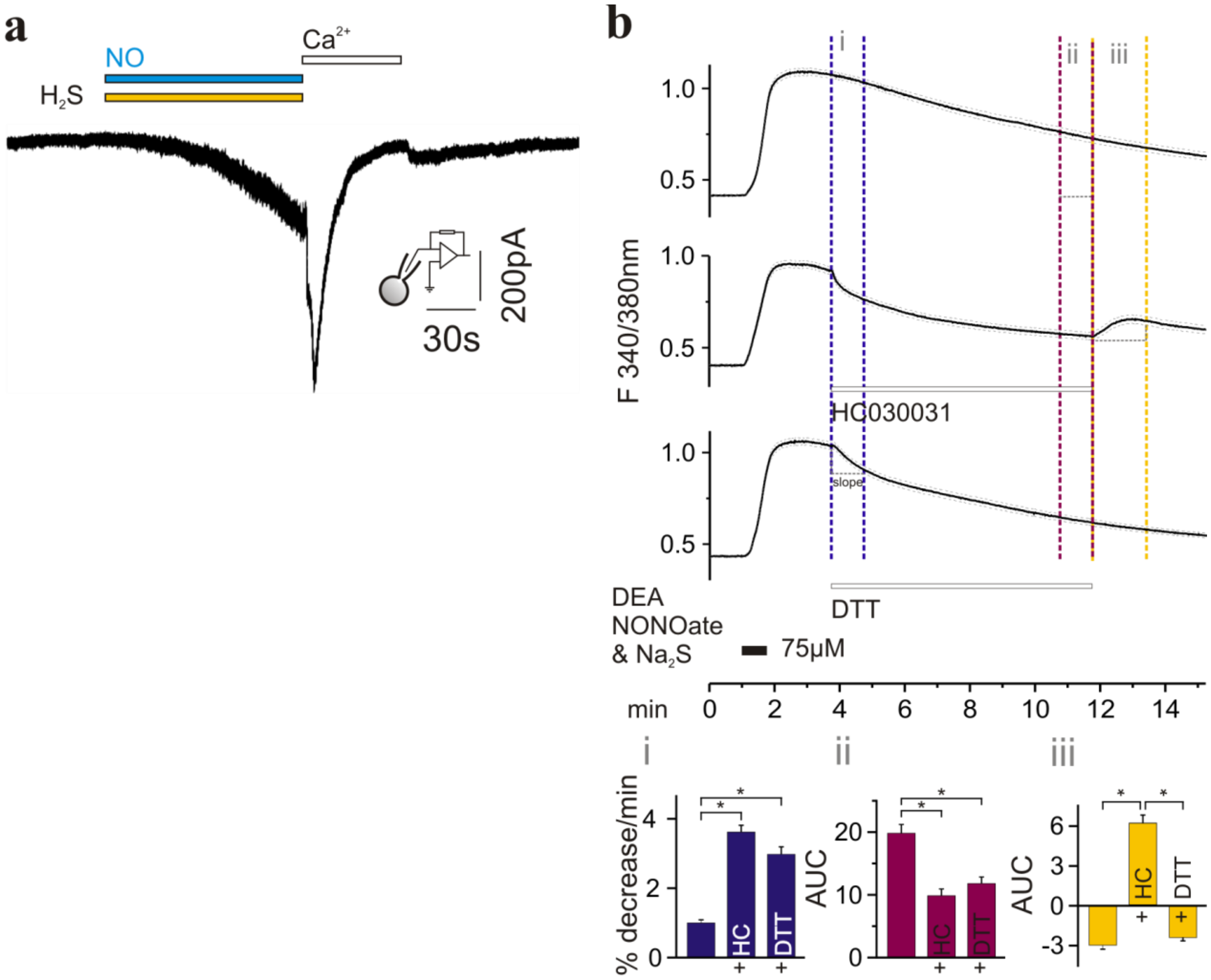

Supplementary Figure 9. Combination of NO and H_2S activates TRPA1 channels

a) NO + H_2S-evoked inward currents in CHO cells expressing mTRPA1 could be strongly potentiated by switching from calcium free to external solution containing 2 mM calcium**. b)** HC030031 and dithiothreitol (DTT) had similar effects on NO + H_2S-induced calcium transients as on transients evoked by AS in DRG neurons: NO + H_2S (75 µM each, 45 s)-evoked increases in intracellular calcium are slow in reversal (upper lane) but can be accelerated by application of HC030031 (50 µM, 10 min, middle lane) or DTT (5 mM, 10 min, lower lane) measured as the percentage of decrease within the first minute of HC/DTT application in (i). This results in a lower area under the curve of intracellular calcium calculated for the tenth minute (ii) after NO and H_2S application in cells treated with HC030031 or DTT. A rebound increase of intracellular calcium was observed upon washout of HC030031, but not DTT (iii), suggesting that DTT deactivated TRPA1, while HC just impeded further calcium entry (all ANOVA following HSD post hoc tests; * $p < 0.001$ each; n = 101 responding DRG neurons per group; all error bars represent SEM).

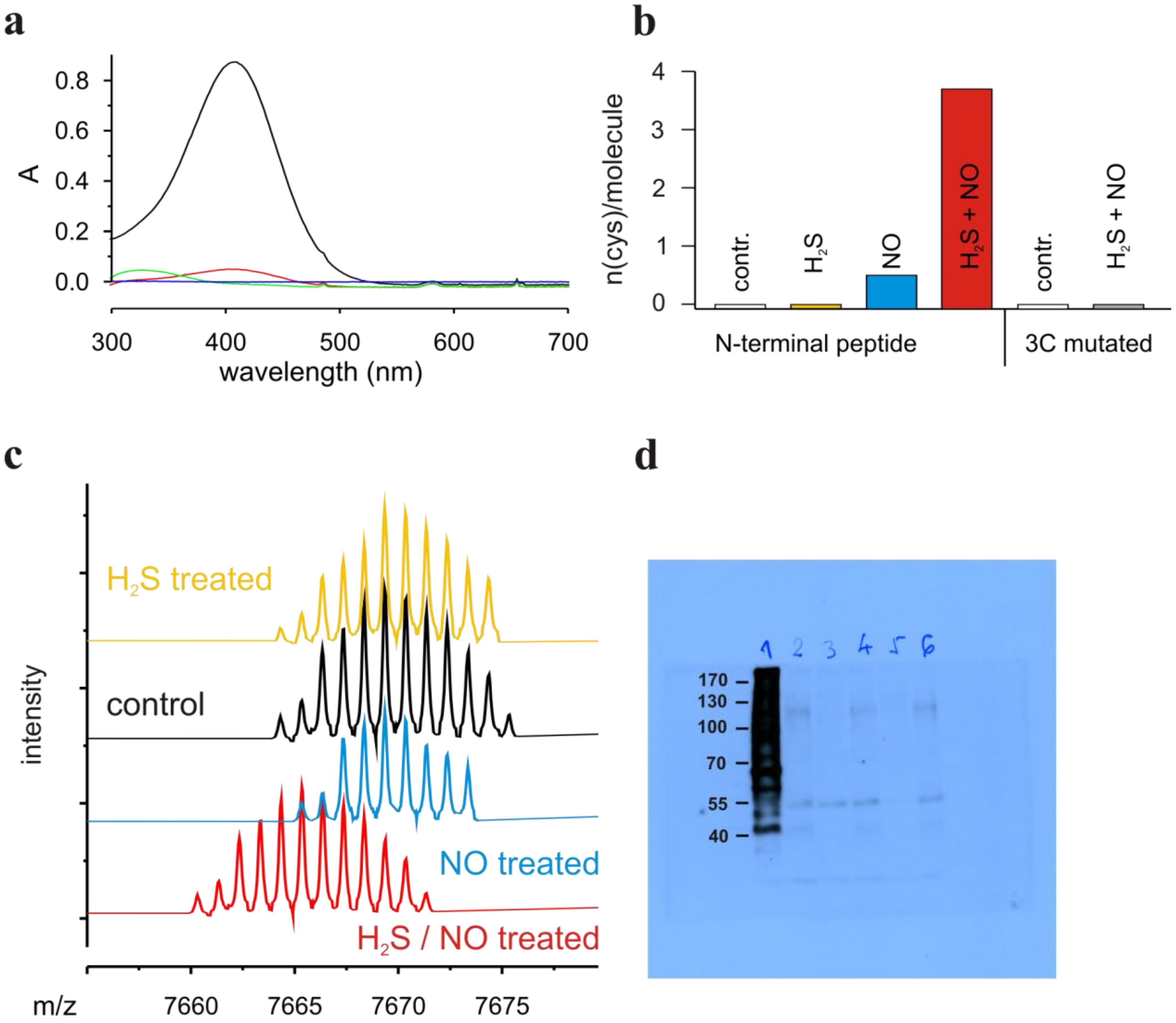

Supplementary Figure 10. HNO generated from NO and H_2S induces disulfide formation in TRPA1

a) UV-vis analysis on hTRPA1 N-terminus treated with NO, H_2S or combination of both under hypoxic conditions (in order to minimize artifactual oxidation), then subsequently purified, treated with IA, then with DTT, cleaned on Micro Bio-Spin® Columns with Bio-Gel® P-6 (Bio-Rad) and finally treated with Ellman's reagent to measure the formation of yellow 3-thio-6-nitrobenzoate at 412 nm. Formation of yellow product was only observed when native polypeptide was treated with combination of NO and H_2S. **b**) Approximately 3.8 moles of cysteine residues per molecule were found to be modified by the combination of NO and H_2S implying formation of two disulfide bonds. The same experiments were also performed with hTRPA1-3C (originally C621S, C641S and C665S) mutated N-terminus. Mutated N-terminus remained unchanged, confirming essential role of the three cysteines (right). **c)** Deconvoluted mass spectra of synthetic, 64 amino acid-long peptide without or with treatment with NO, H_2S or combination of both, showing the mass shift for *m/z* 4 only when combination was used and confirming formation of two disulfide bonds observed for AS. **d)** Detection of intramolecular disulfides (1-2), *S*-nitrosothiols (3-4) and *S*-sulfhydration (5-6) in TRPA1 isolated form DRG neurons (lanes 1, 3, 5) or DRG neurons treated with 2 mM combination of oxamic acid and L-NMMA for 12 h (lanes 2, 4, 6). As the signal in the lane 1 was so intense, overexposed film is presented here in order to visualize potential modifications in the other lanes.

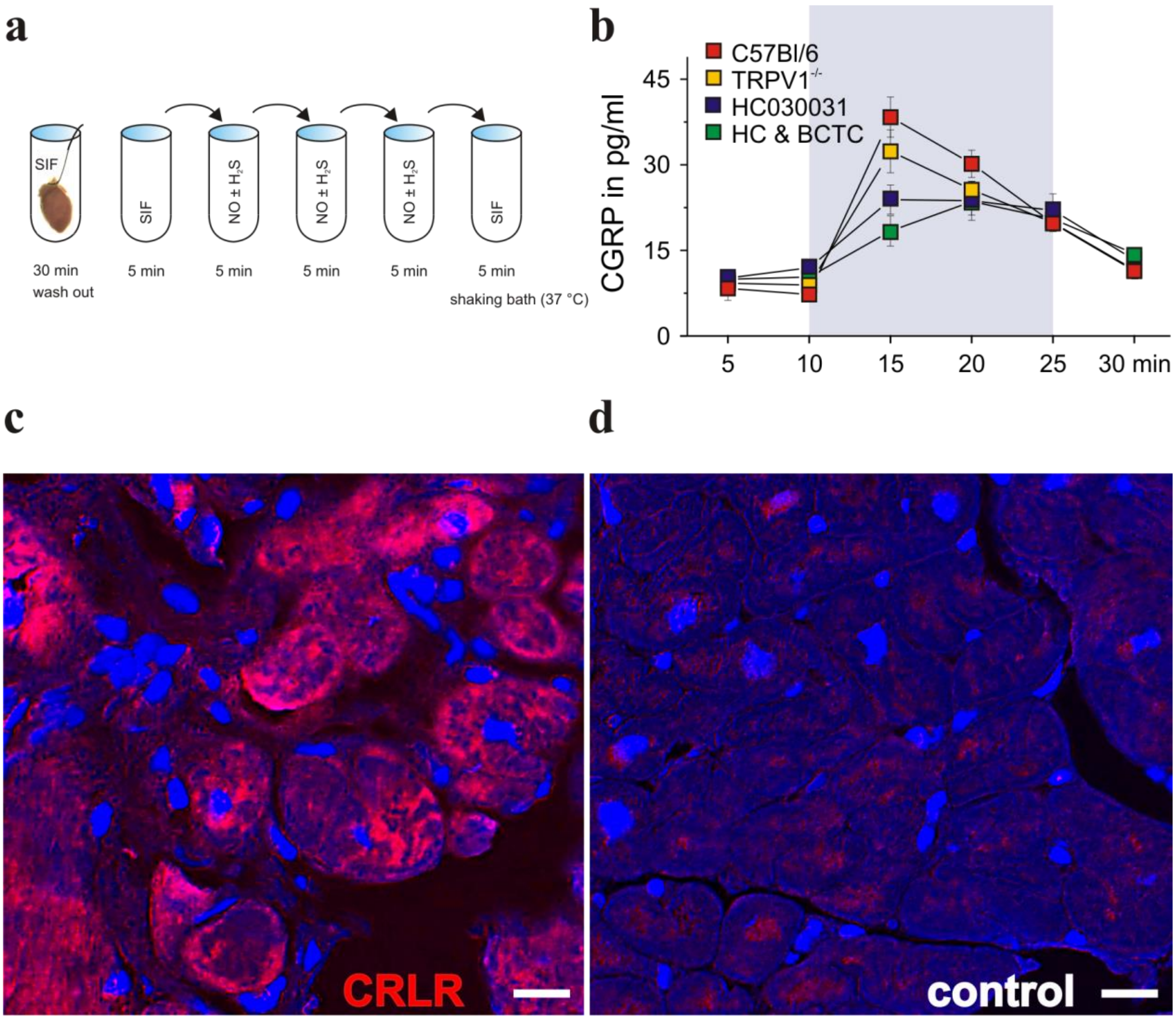

Supplementary Figure 11. Detection of CGRP receptor expression in human cardiomyocytes and CGRP release from isolated mouse heart

a) Scheme for experiments stimulating CGRP release from isolated mouse heart. CGRP content of the solutions is determined by EIA. **b)** Combined application of NO and H_2S (250 nmol per heart) stimulated CGRP release to a similar extent in C57Bl/6 and congenic TRPV1 knockout mice ($p = 0.179$). While responses could be reduced by the TRPA1 blocker HC030031 ($p = 0.002$; C57Bl/6 vs. HC), additional block of TRPV1 receptors by BCTC did not diminish stimulated CGRP release further ($p = 0.36$ HC vs. HC & BCTC, ANOVA LSD post hoc tests; $n = 6$; error bars represent SEM). **c-d)** Immunofluorescence images of human cardiomyocytes in crosssections stained for CRLR **(c)** show membranous and cytoplasmic reactivity that spares myofibers; **(d)** negative control on sections of the same patient (scale bars = 25 µm).

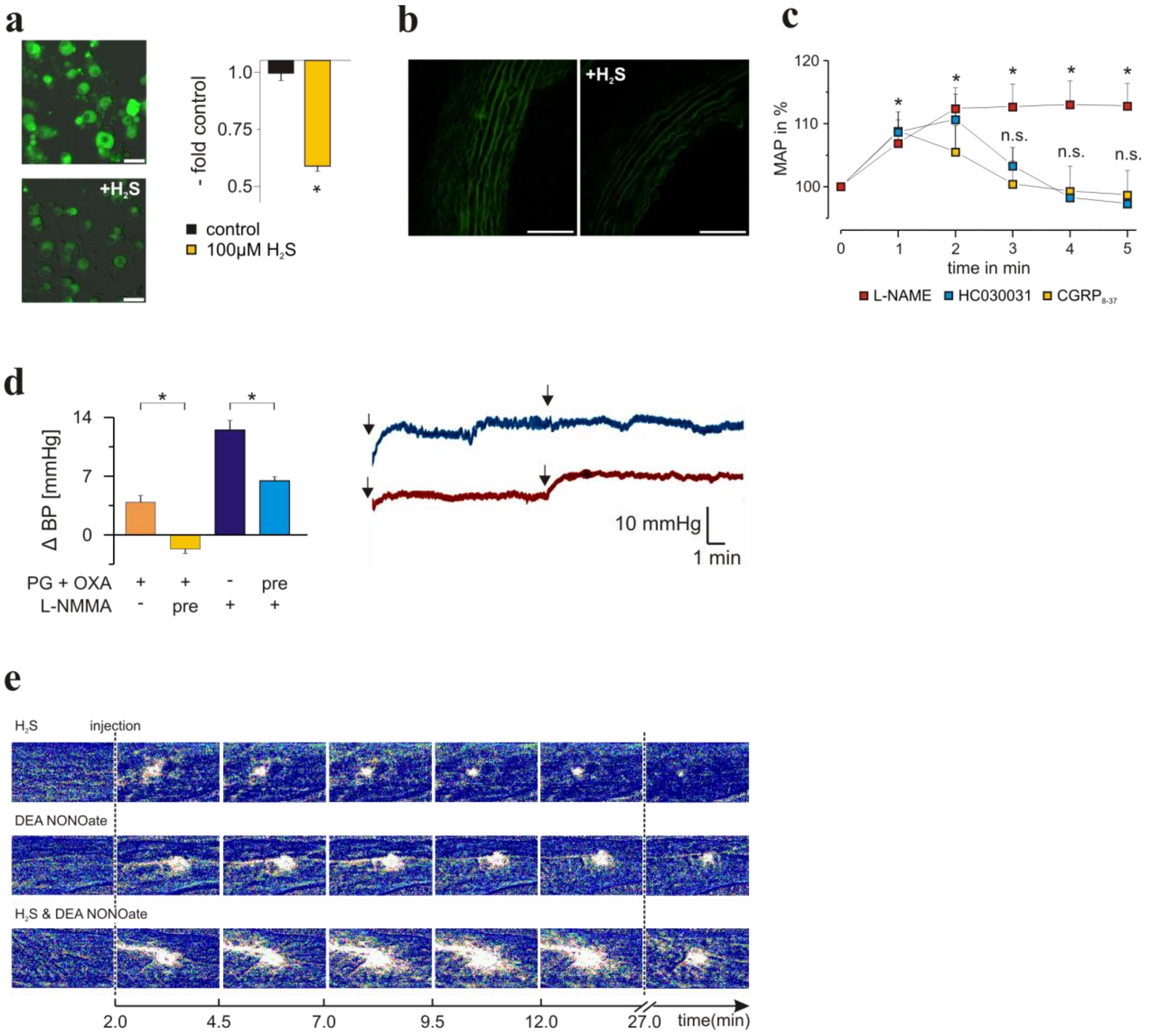

Supplementary Figure 12. H_2S decreases NO levels in the cells and induces vasodilatation *via* HNO-TRPA1-CGRP

a) Left: Confocal microscopy images of DAF-2FM-DA loaded DRG neurons without or with co-administration of 100 µM H_2S. Exposure to 100 µM H_2S leads to significant drop of intracellular NO levels as NO is consumed in the reaction with H_2S to give HNO (scale bar = 30 µm). **Right:** Quantification from the obtained micrographs ($p<0.05$; t-test, n = 100 DRG neurons per group; mean ± SEM). **b)** Aorta rings loaded with DAF-FM diacetate (1 h) were treated without or with 100 µM H_2S for 1h. An obvious decrease in green fluorescence could be observed in H_2S treated aortas, again confirming that reaction of H_2S and NO could occur in non neuronal tissues (scale bar = 100 µm). **c)** Changes of rat mean arterial pressure upon i.v. injection of L-NAME (1.3 mg), HC030031 (15 nmol) and $CGRP_{8\text{-}37}$ (50 µg kg^{-1}) show that all three induce almost the same increase in blood pressure, confirming the existence of an endogenous path for TRPA1-CGRP-induced regulation of systemic blood pressure (ANOVA, LSD post hoc test; n = 5, * $p < 0.05$, mean ± SEM). **d**) Changes of MAP in mice induced by the inhibitors of H_2S and NO production. Oxamic acid (OXA; 6 µg g^{-1} body weight) and propargylgycine (PG) each were injected intravenously and after 10 min of L-NMMA (6 µg g^{-1} body weight) was followed, inducing a rise of MAP (red trace). The reverse experiment was performed with first injection of L-NMMA, inducing a marked MAP rise, followed by OXA+PG (blue trace) which caused no further MAP rise. Changes of the MAP are represented in the histogram on the left and the representative traces of blood pressure monitoring on the right (ANOVA HSD post hoc test; * $p \leq 0.001$; n = 6 each; mean ± SEM). **g)** Complete series of laser Doppler images taken before and every 2.5 minutes after double-blinded intracutaneous injection of H_2S (0.35 µmol), DEA NONOate (0.23 µmol) or combination of both to human volunteers' volar forearms.
